# Bile acid derivatives from gut microbiota promote GBPs-mediated activation of caspase-4/11 by LPS through *lncRNA57RIK*

**DOI:** 10.7150/ijbs.97059

**Published:** 2024-10-28

**Authors:** Yunhuan Gao, Jianmei Yue, Fushuang Ha, Ya Wang, Rong Wang, Xiaorong Yang, Junqi Zhang, Xinqi Liu, Yuan Zhang, Tao Han, Rongcun Yang

**Affiliations:** 1 Translational Medicine Institute, Affiliated Tianjin Union Medical Center of Nankai University, Tianjin 300071, China.; 2 State Key Laboratory of Medicinal Chemical Biology, Nankai University, Tianjin 300071, China.; 3 Department of Immunology, Nankai University School of Medicine; Nankai University, Tianjin 300071, China.; 4 The Third Central Clinical College of Tianjin Medical University, Tianjin 300170, China.; 5 College of life Science, Nankai University, Tianjin, China, Tianjin 300121, China.; 6 Tianjin Union Medical Center, Tianjin Medical University, Tianjin 300270, China.

**Keywords:** macrophages, * lncRNA57RIK*, caspase-4/11, guanylate-binding protein 1, sphingosine-1-phosphate receptor 2

## Abstract

Lipopolysaccharide (LPS) mediated caspases-4 (humans) and caspase-11 (rodent) (caspase-4/11) signaling can cause maturation of inflammatory cytokine IL-1β and cellular pyroptosis in the macrophages through guanylate-binding proteins (GBPs). However, how caspase-4/11s bind with GBPs together to activate caspase-4/11 by LPS remains elusive. We here found that BA derivatives from gut microbiota can regulate sensitivity of macrophages to LPS and Gram-negative bacteria through *lncRNA57RIK*. BA derivatives such as deoxycholic acid (DCA) could induce *lncRNA57RIK* expression through sphingosine-1-phosphate receptor 2 (S1PR2) in the macrophages of mice and humans. Both murine and human *lncRNA57RIK* knockout (KO) macrophages did not produce immune response(s) to LPS or gram negative bacteria. *LncRNA57RIK* KO mice had also reduced inflammatory responses to LPS or *Salmonella* typhimurium (*S.* T) infection. Mechanistically, *lncRNA57RIK* could bind intracellular proteases caspase-4/11 with GBP1 together in the macrophages of human and mice to cause LPS-mediated activation of caspase-4/11. Thus, BA derivatives from gut microbiota promote GBPs-mediated activation of caspase-4/11 by LPS through *lncRNA57RIK*.

## Introduction

Lipopolysaccharide (LPS, endotoxin) mediated caspases-4 (humans) and caspase-11 (rodent) (caspase-4/11) signaling can cause maturation of inflammatory cytokine IL-1β and cellular pyroptosis in the macrophages. Both the pyroptosis and IL-1β are essential to an efficient immune response against various bacteria such as *Shigella flexneri*, *Salmonella* Typhimurium (*S.* T), and enterohemorrhagic *E. coli* (EHEC) 1-3. In addition, active caspase-4/11 can also cleave Gasdermin-D (GSDMD), which form GSDMD pore to cause NLRP3 activation and IL-1β maturation in macrophages 4. Notably, caspase-4/11 activation in macrophages with LPS or Gram-negative bacteria requires the expression of interferon (IFN)-inducible guanosine triphosphate (GTP)ases, such as guanylate-binding proteins (GBPs) and/or immunity-related GTPases (IRGs) 5-8. Indeed, LPS can mediate assembly of GBPs on the surface of bacterium such as *Salmonella* in the cytoplasm (or on LPS-rich membrane interfaces) 9, 10. A complex formed by GBP-LPS can promote the recruitment of caspase-4/11 and subsequently transfer LPS onto caspase-4/11, triggering its activation 9. Recent studies have shown that the macrophages lacking GBPs impair caspase-4/11 activation and attenuate pyroptosis 5. However, it is unclear how the caspase-4/11s bind with GBPs together to activate caspase-4/11 by LPS.

Long noncoding RNAs (LncRNAs) exert an important role in mediating interaction of intracellular proteins with their substrates. They are involved in many important biological phenomena 11, 12. LncRNA overexpression, deficiency or mutation has been associated with many human diseases 13. Studies have suggested that the interaction of some intracellular proteins with their substrates needs lncRNA 14-16 such as lncRNA DRAIC inhibits prostate cancer progression by interacting with IKK to inhibit nuclear factor (NF)-κB activation 16.

Gut microbiota such as bacteria, fungi and viruses, which inhabits in the gastrointestinal tract, contributes to the regulation of host immune responses through their metabolites such as bile acid (BA) derivatives 17. The primary BAs cholic acid (CA) and chenodeoxycholic acid (CDCA) are generated in the liver. These primary CDCA or CA can be conjugated to one or more amino acids such as alanine, arginine and aspartate to form conjugated BAs 18. Then BAs are deconjugated and converted into secondary BAs deoxycholic acid (DCA) and lithocholic acid (LCA) by gut microbiota in colon. In addition, a range of derivatives such as 3-oxoLCA, 7-oxoCDCA are also generated by gut bacteria 19-21. These different BA derivatives can exert different roles in immune cells through different receptors such as cellular membrane receptors such as G-protein BA receptor 1 (GPBAR1) known as TGR5, and nuclear receptors such as farnesoid X receptor (FXR), pregnane X receptor (PXR) and sphingosine-1-phosphate receptor 2 (S1PR2) 17. Generated secondary BA deoxycholic acid (DCA) and lithocholic acid (LCA) by gut microbiota could induce inflammatory macrophage 22-24 such as that DCA induced activation of the NLRP3 (NOD-like receptor thermal protein domain associated protein 3) inflammasomes in the macrophages to promote inflammation in cholestasis-associated sepsis 22. Notably, through BA receptors Takeda G protein-coupled receptor 5 (TGR5) and farnesoid X receptor (FXR), BA metabolites were essential to maintain tolerant phenotypes of the macrophages 25-27. The release of IL-1β in FXR and TGR5-deficient bone marrow (BM)-derived macrophages was significantly reduced upon *E.coli* infection 28. However, in addition of FXR and TGR5 receptors, the macrophages also expressed other multiple receptors such as S1PR2 29-31. Activation of these receptors by BAs might produce different effects on the differentiation and function of macrophages.

We here found that secondary BA metabolite DCA generated by gut microbiota could promote production of mature IL-1β (mIL-1β) and pyroptosis of macrophages through S1PR2 mediated* lncRNA57RIK* expression. We also demonstrate that DCA mediated *lncRNA57RIK* can induce the binding of caspase-4/11 with GBPs to activate caspase-4/11 by LPS, and also is necessary for Gram-negative bacterium mediated infection.

## Materials and Methods

Reagents and oligoes used in this paper were listed in supplementary Table S1.

### Mice and cell lines

*LncRNA57Rik* deficient mice on a C57BL/6J background were generated by the Model Animal Research Center of Nanjing University (Nanjing, Jiangsu, China) using CRISPR-Cas9 system as previously reported by us 32, 33. Cas9 mRNA and sgRNA were co-injected into zygotes, sgRNA direct Cas9 endonuclease cleavage in upstream of E1 and downstream of E4, and create a DSB (double-strand break). The breaks were repaired by non-homologous end joining (NHEJ), and resulted in deletion of 5730403I07Rik gene (*LncRNA57Rik*). Caspase1/11-/- mice were from Prof. Shao, National Institute of Biological Sciences, Beijing; *TGR5*-/- was from Prof. Meng, Pasteur Institute, shanghai; *PXR* -/- mice were from Institute of Model Animal, Wuhan University; C57BL/6 and B6.SJL-CD45a (Ly5a) (CD45.1) mice were purchased from the Animal Research Center of Nanjing University (Nanjing, Jiangsu, China). All mice were maintained under specific pathogen-free (SPF) conditions in the Animal Center of Nankai University.

Human embryonic kidney cell line HEK 293T cells were obtained from the American Type Culture Collection, and cultured in DMEM cell culture medium containing 10% FBS, 1% penicillin, and streptomycin.

### Mouse models

For *Salmonella* Typhimurium (*S.* T*.*, ATCC14028) infection, *Salmonella* infection model was performed according to the previous method 34. Briefly, mice were withdrawn from water and food for 4 hours before oral gavage treatment with 7.5 mg of streptomycin. After streptomycin treatment, mice were withdrawn from water and food again and then infected with *S.* T. (200 cfu). Mice were weighed every other day for the determination of percent weight change. This was calculated as: % weight change = (weight at day X-day 0 / weight at day 0) × 100. Mice were sacrificed at the indicated days for histological study. Representative colon and lung tissues were embedded in paraffin for hematoxylin/eosin (H&E) staining or in OCT compound (Tissue-Tek, Sakura, Torrance, CA) for immuno-staining. Lung, liver, and spleen were collected and then homogenized for 2 min in PBS with metal beads by using a TissueLyser II apparatus (Qiagen). CFUs were quantified by plating lysates onto LB agar, followed by incubation overnight. Histological scores were assessed according to our previously reported methods 35, 36. For chronic toxicity of LPS, mice were intraperitoneally injected with 20mg/kg LPS (O111:B4), and then serum IL1β and IL18 concentration were detected. For acute toxic experiment, mice were injected with 52mg/kg LPS (O111:B4), and then survival (Time to moribund) were detected.

For animal model of bile duct ligation (BDL), mice were anesthetized with 45 mg/kg pentobarbital. The abdominal cavity was opened from the midline of the abdomen. The common bile duct was ligated twice with 1-0 silk suture, and the bile duct was cut between ligations. Sham mice were subjected to open abdominal surgery only without BDL.For bone marrow cell (BMC) transplanted experiments (BMT), BMCs collected from wt or *lncRNA57RIK-/-* mice were injected into different recipient mice, which were irradiated (800 cGy, a single dose) using a Shepherd Mark I Cesium Irradiator (J.L. Shepherd and Associates). After 3 weeks,* S.* T infection and BDL were performed in these recipient mice.

### Preparation of macrophages

For human monocytes derived macrophages (HMDM), primary human peripheral blood mononuclear cells (PBMCs) were isolated by Ficoll-Hypaque density gradient centrifugation. CD14^+^ magnetic isolation kit was used to isolate monocytes/macrophages following the manufacturer's instructions. Monocytes/macrophages were cultured in DMEM with 10% FBS, 50 ng/ml human M-CSF (PeproTech), and 1% penicillin/streptomycin for 4 d, and then used for experiments. For THP1 derived macrophages, THP-1 cells were treated 24 h with 100 ng/mL PMA. For mouse bone marrow derived macrophages (BMDMs), BMDMs were obtained from bone marrow of the tibia and femur, and cultured in DMEM with 10% FBS, 20 ng/ml mouse M-CSF (PeproTech)and 1% penicillin/streptomycin for 6 d and then replated and used for experiments. For macrophages from peritoneal cavity of mice, macrophages were generated in the peritoneal cavity of mice by intraperitoneally injected with 4 mL of 3% thioglycollate medium. After four days, 5 mL of cold phosphate-buffered saline (PBS) containing 3% FBS was injected into the peritoneal cavity. Following this injection, a gentle massage was performed, and peritoneal fluid was subsequently isolated. Next, cells derived from the peritoneal washing fluid were seeded at 2 × 10^6^ in RPMI containing 10% FBS. Nonadherent cells were removed 4 h after seeding by extensive washing with medium.

### Macrophage stimulation

For macrophages stimulation, to induce canonical inflammasome, macrophages were primed overnight with 100 U/mL IFN-γ followed by priming with 2 μg/mL LPS for 4 h or followed by treatment with 5 µM Nigericin (MedChemExpress) or transfection with 5μg/mL of Flagellin (AdipoGen Life Sciences), 2 μg/mL LPS (Sigma) using Dotap chloride transfection reagent (Selleck) for 30 min (Nigericin) or 2 h (Flagellin and LPS). THP-1 cells were treated 24 h with 100 ng/mL PMA (selleck) before overnight stimulation with 100 U/mL IFN-γ (PeproTech) followed by infection. Then supernatants were analyzed for IL-1β by ELISA and lactate dehydrogenase (LDH) by LDH detecting Kit. CDCA (Chenodeoxycholic acid, 50μM), TCA (taurine-conjugated cholic acid, 50μM), DCA (100μM) and LCA (50μM) were used in these experiments except for specific indication. FXR (Farnesoid X receptor) antagonist 1 (50μM), TEI-9648(VDR (Vitamin D receptor) inhibitor, 100nM), DHODH-IN-3(ROR (Retinoic acid-related orphan receptor) γt inhibitor, 2μM), Larsucosterol (LXR (Liver X receptor) inhibitor, 10μM), Resveratrol (PXR (Pregnane X receptor) inhibitor, 50μM), SBI-115(TGR5 (Takeda G protein-coupled receptor 5) inhibitor, 100μM), CINPA1(CAR (Constitutive androstane receptor) inhibitor, 1μM), JET-013(S1PR2 inhibitor, 100μM), MRGPRX4 modulator-1(MRGPRX4 (Mas-related G protein-coupled receptor X4) inhibitor, 100nM), CMC2.24(Ras inhibitor, 50μM), SCH772984 (ERK (Extracellular-signal-regulated kinase) inhibitor, 300nM), Wortmannin (PI3K (Phosphatidylinositol 3-kinase) inhibitor, 50nM), JNK-IN-8 (JNK (C-Jun N-terminal kinase) inhibitor, 400nM), and Perifosine (AKT (Protein kinase B) inhibitor, 50μM) were used in these experiments.

### *Salmonella* infection on macrophages

For *Salmonella* infection**,** macrophages were primed with 10 ng/mL of IFNγ (Peprotech) for 16 hours prior to being infected. Salmonella were cultured overnight (OD600 =1.5-1.8), collected by centrifugation and resuspended in DMEM. Salmonella was added to cells in 96-well plates (~50,000 cells per well) at a multiplicity of infection (MOI) of 50 and incubated for 30 min at 37°C. Denaturalized bacteria were removed with three washes of warm DMEM, and cells were incubated with DMEM containing 100µg/mL gentamicin for 1 h to kill extracellular bacteria. DMEM containing 10µg/mL gentamicin and 10% FBS was used for the rest of the experiment.

### MicroRNA transfection, preparation of plasmids, shRNA or *lncRNA57RIK* lentiviruses construction and transduction

For microRNA transfection, the macrophages were transfected with microRNAs using HiPerFect transfection reagent (Qiagen, Valencia, CA, USA) according to the manufacturer's instructions. For preparation of plasmids, the sequences or fragments of mouse caspase11, mouse GBP1, human caspase4, human GBP1 and mouse/human *lncRNA57RIK* were amplified using PCR methods. The PCR products were cloned into the pcDNA™3.1/V5-His TOPO® TA plasmid (Invitrogen). After sequencing, plasmid constructions were used to transfect HEK 293T cells. For shRNA lentivirus construction and transduction, a short hairpin RNA (shRNA) target sequence was chosen by BLOCK-iT™ RNAi Designer (Invitrogen). The shRNA or *lncRNA57RIK* constructs were made using pGreenPuro™ cloning and expression lentivector kit (System Biosciences Inc.) according to the manual. The control NC was luciferase control RNA from the kit. For packaging lentivirus particles, the shRNA or *lncRNA57RIK* lentivector together with pMD2.G and psPAX2 packaging plasmids were co-transfected into 293T cells. The macrophages were infected with the lentiviral supernatants in the presence of 8μg/ml polybrene (Millipore) by centrifugation and then cultured with complete medium for 24 hours.

### RNA-seq analysis

For RNA-seq analysis, BMDMs were generated and then treatment with 100 µM DCA for 24h. RNA-seq libraries were prepared with the TruSeq sample preparation kit while total RNA was extracted using an RNeasy mini kit. Using STAR aligner (v2.5.0c), all of the reads were mapped to the mouse reference genome (GRCm38/mm10). The mean read insert sizes and their standard deviations were calculated using Picard tools (v.1.126) (http://broadinstitute.github.io/picard/) while alignments were guided by a Gene Transfer File (Ensembl GTF version GRCm38.74). Using BEDTools (v2.17.0) and bedGraphToBigWig tool (v4), the Read Per Million (RPM) normalized BigWig files were generated. Through HTSeq (v0.6.0) normalized based on their geometric library size factors using DESeq2 (v3.0), read count tables were generated. Deferential Expression (DE) analyses were performed.

### *LncRNA57RIK* deletion in macrophages

First, the target gene sequence was analyzed. Suitable target sites were screened, and then one sgRNA for each target site was designed. According to the designed sgRNA sequence, oligo DNA was synthesized. The empty vector was digested to obtain linearized plasmid. Oligo DNA was mixed with linearized empty vector and ligated with T4 ligase and connected overnight at 16°C. The verified plasmid was amplified and cultured, and then plasmid was extracted. THP-1 cells were seeded in 12-well plates at a density of 100,000 cells per well. After 24 h, the cells were transiently transfected with 4 μg Cas9 plasmid using electrotransfection. After 24 h of transfection, puromycin was added with a final concentration of 3 ug to the culture dish for screening. After 3 days, the cells were diluted to a density of 1-2 cells/200 ul in 40 mL of complete growth medium, cells were plated in a 96-well plate. After 5 days, the 96-well plate was observed under an optical microscope and marked the wells containing single cells. After the cell density reaches 70%~90%, the cell genome is extracted and amplified with the PCR primers, and the amplified products are sequenced.

### Generation of caspase 4 and GBP1 KO cell lines

Caspase 4 and GBP1 knockout THP-1 cell lines were generated by using a mix of sequence-specific CRISPR RNA (crRNA), transactivated crRNA (tracrRNA), and recombinant Alt-R S. pyogenes Cas9 (IDT). The mixed crRNA and tracrRNA (1 µM) were annealed (heated at 95°C for 5 min and cooled to room temperature) and mixed with 1 µM Alt-R Cas9 and incubated at room temperature for 5 min. HiPerFect Transfection Reagent (QIAGEN) was then added, and the mixture was incubated for 20 min at room temperature and cells were transfected. After incubation at 37°C and 5% CO2 for 2 days, single clones were generated by serial dilution and the desired knockouts were screened by T7 endonuclease I assay, verified by sequencing, and confirmed by Western blotting. The crRNAs used listed in (Table S1).

### Isothermal titration calorimetry

Isothermal titration calorimetry (ITC) was conducted with a MicroCal iTC200 instrument. To get concentration for isothermal titration, lyophilized RNA samples were prepared in the titration buffer, renatured at 95^o^C for 2min, 4^o^C for 2min and 25℃ for 20min, and then diluted; Human and caspase 4 proteins were dialyzed in the titration buffer containing 150 mM NaCl, 2 mM MgCl_2_ and 20 mM Tris-HCL, pH 7.4. Using software origin 7.0 based on the 'One Set of Sites' fitting model, acquired calorimetric titration data were analyzed.

### Cytosolic and nuclear fractionation

The cells were first incubated with hypotonic buffer (25 mM Tris-HCl, pH 7.4, 1 mM MgCl2, 5 mM KCl) on ice for 5 min, and then an equal volume of hypotonic buffer containing 1% NP-40 was then added. Each sample was left on ice for another 5 min. The supernatants were collected as the cytosolic fraction bycentrifugation at 5000 × g for 5min. The pellets were resuspended in nucleus resuspension buffer (20 mM HEPES, pH 7.9, 400 mM NaCl, 1 mM EDTA, 1 mM EGTA, 1 mM DTT, 1 mM PMSF) and incubated at 4 °C for 30 min. After centrifugation at 12,000g for 10 min, nuclear fraction was collected after removing insoluble membrane debris.

### Cell isolation and flow cytometry

Cell isolation and flow cytometry were performed according to our reported protocol 33, 37, 38.

### Immunostaining and RNA-FISH

Immunostaining and RNA fluorescence *in situ* hybridization (RNA-FISH) were performed according to our reported protocol 33, 37, 38. **H & E staining.** For hematoxylin/eosin (H&E) staining, previously reported methods were used in this experiment 33, 37, 39. **RNA extraction and qRT-PCR.** RNA extraction and qRT-PCR were analyzed according to ore previously reported methods 33, 37. **Northern blot, Western blot and Immunoprecipitation.** For northern blot and Western blot, previously reported methods were used in this study 33, 37, 39. **RNA Immunoprecipitation (RIP) and UV-RIP**. RNA immunoprecipitation was performed according to previously reported protocol 33, 37. **RNA-protein pull-down analyses**. For RNA-protein pull-down analyses, previously reported methods were used in this study^33^, ^37^. **Gene binding motif analyses.** Sequence logo of gene binding motif was obtained used the MEME software (https://meme-suite.org/meme/) (lower). NCBI database was used to perform sequence comparison to find out homologous sequences.

### Statistical analyses

Two side Student's t-test and ONE-way ANOVA Bonferroni's Multiple Comparison Test were used to determine significance. The statistical significance of the survival curves was estimated using Kaplan and Meier method, and the curves were compared using the generalized Wilcoxon's test. The data from patient samples were analyzed by a Mann-Whitney U test. These were performed by GraphPad Prism 5 software (GraphPad Software). A 95% confidence interval was considered significant and was defined as p <0.05.

## Results

### BA metabolite DCA promotes IL-1β maturation and pyroptosis of human macrophages

**Figure 1:**
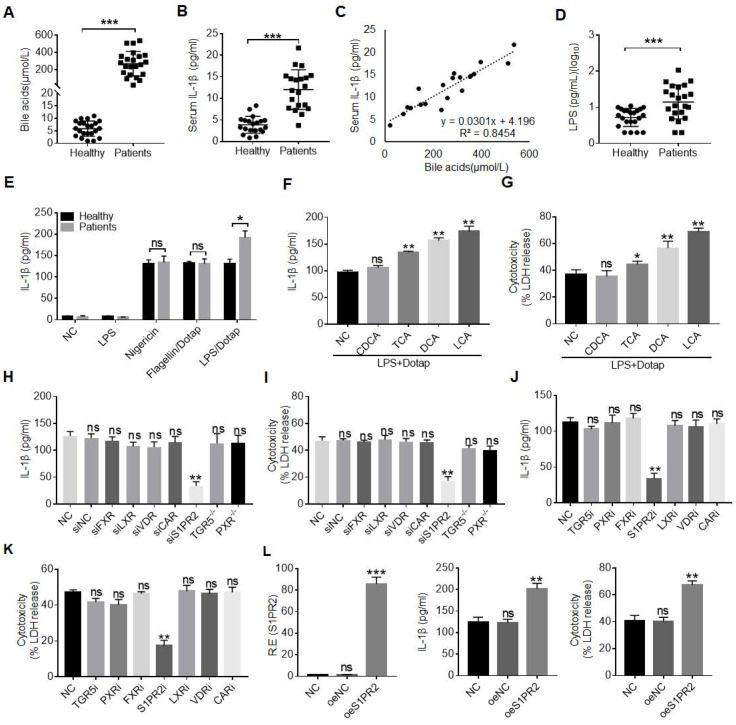
**BA derivatives promote caspase-4 mediated IL-1β maturation and pyroptosis of macrophages. (A)** ELISA of total bile acids in the sera of healthy volunteers and patients with cholestasis (n=22). **(B)** ELISA of IL-1β in the sera of healthy individuals and patients with cholestasis (n=22). **(C)** Correlation analyses between serum IL-1β and bile acids in the patients with cholestasis using correlation and regression. R2, Pearson correlation coefficient. **(D)** Analyses of LPS in sera of healthy individuals and patients with cholestasis (n=22).** (E)** ELISA of IL-1β in the supernatants of the monocytes/macrophages of healthy individuals and patients with cholestasis after exposure to different stimulators. Monocytes/macrophages were isolated using flow cytometry. **(F)** ELISA of IL-1β in the supernatants of the human monocytes/macrophages after exposure to chenodeoxycholic acid (CDCA), TCA, DCA and LCA, and then stimulated by LPS with Dotap. **(G)** Analyses of LDH in the supernatants of the human monocytes/macrophages after exposure to CDCA, TCA, DCA and LCA, and then stimulated by LPS with Dotap. **(H)** ELISA of IL-1β in the supernatants of the monocytes/macrophages after silencing or knocking out BA receptors. **(I)** Analyses of LDH from the monocytes/macrophages after silencing or knocking out BA receptors. **(J & K)** ELISA of IL-1β (J) and analyses of LDH (K) from the monocytes/macrophages treated with different BA receptor inhibitors. **(L)** ELISA of IL-1β and analyses of LDH in the supernatants of monocytes/macrophages infected using BA receptor S1PR2 lentiviruses. Human macrophages were pretreated with DCA, activated with LPS and followed by stimulation with LPS /Dotap in (H-L). R. E, relative expression; a Mann-Whitney U test used in (A, B and D); ONE-way ANOVA Bonferroni's Multiple Comparison Test used in (F-L); Student's t-test in (E); *P < 0.05, **P < 0.01, ***P < 0.001, NS, not significant.

The patients with cholestatic jaundice often suffer from infections and sepsis with Gram-negative bacteria such as* E. coli.* 22, 40, 41, implying that cholestatic jaundice can promote inflammation. Indeed, there not only existed higher levels of BAs but also inflammatory cytokine IL-1β, consistent with other reports (Figure 1A, B) 22, 42. Notably, there existed a positive relationship between the levels of BAs and inflammatory cytokine IL-1β (Figure 1C). In addition, higher levels of LPS, which might partially explain the increased mortality of sepsis under the conditions of cholestasis 22, 40, 41, were also observed (Figure 1D). However, it was unclear how LPS caused the increased mortality of sepsis under the condition of cholestasis 22. Inflammasome activation was shown to be a pivotal player in sepsis despite the debate over the role of IL-1β in sepsis-associated mortality 43, 44. Surprisingly, we found that LPS with dotap, noncanonical inflammasome caspase-4/11 ligand, but not LPS with nigericin (NLRC3 ligand) 45, or LPS with flagellin (NLRC4 ligand) 46 could induce more IL-1β production in the monocytes/macrophages isolated from peripheral blood monocytes/macrophages of patients with cholestasis than those from healthy individuals (Figure 1E), implying that BAs promote sensitivity of monocytes/ macrophages to caspase-4/11 ligands. Indeed, data showed that not only the conjugated BAs taurocholic acid (TCA) but also secondary BAs deoxycholic acid (DCA) and lithocholic acid (LCA) but not primary BA chenodeoxycholic acid (CDCA) could promote maturation of IL-β (Figure 1F). Interestingly, BA metabolites such as DCA and LCA, which were generated by gut microbiota, were also higher in the sera of the patients with cholestatic jaundice (Figure S1A and B). The production of mIL-1β often companies with pyroptosis 47, 48, which is a lytic cell death induced by pathogen infection or endogenous challenge 49. Caspase-4/11 ligand LPS with dotap also induced more LDH release in the monocytes/macrophages isolated from patients than those in healthy individuals, indicating that caspase ligands can also induce more pyroptotic monocytes/macrophages in the patients (Figure 1G).

Since our goal was to determine the effects of gut microbiota associated BA derivatives on the macrophages, the secondary bile acids such as DCA generated by gut microbiota was higher in the serum of cholestatic jaundice patients 50 (Figure 1SC). More importantly, the secondary BA DCA but not primary BA chenodeoxycholic acid (CDCA) could promote production of IL-β in the macrophages (Figure 1F). Thus, we next determined what kinds of receptor(s) was involved in DCA mediated production of mIL-1β and pyroptosis. BA metabolites can activate a range of nuclear receptors such as FXR, liver-X-receptor (LXR), pregnane X receptor (PXR) and cell membrane receptors such as TGR5, S1PR2, cholinergic receptor muscarinic 2 and 3 (CHRM2, CHRM3), which can be potentially expressed in the macrophages 29-31. To found a potential receptor(s), which is involved in BA metabolites mediated release of mIL-1β, we screened these receptors via siRNA silencing, inhibitors and/or knockout techniques. Interestingly, data showed that S1PR2 was a common receptor of TCA, DCA and LCA, which is potentially involved in BA metabolites mediated mIL-1β production and pyroptosis (Figure 1H-K and Figure S1D-G). S1PR2 mediated production of IL-1β and pyroptosis by DCA were further confirmed by overexpression of S1PR2 (Figure 1L). Notably, levels of the DCA in the sera of patients with cholestatic jaundice are sufficient to activate S1PR2 *in vitro* (Figure S1C). Thus, BA metabolite DCA can promote production of mIL-1β and pyroptosis of monocytes/macrophages via S1PR2.

### DCA mediated IL-1β maturation and pyroptosis is via S1PR2 induced *lncRNA57RIK* in human macrophages

**Figure 2:**
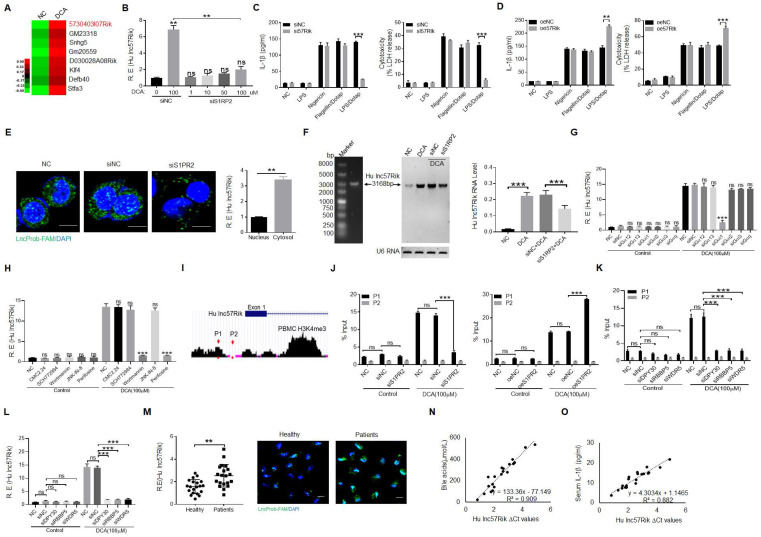
**BA derivative DCA induces *hulncRNA57RIK* expression to regulate IL-1β maturation. (A)** Heatmap showing high expression of genes in the macrophages after exposure to DCA using RNA-seq. **(B)** qRT-PCR of *lncRNA57RIK* in S1PR2 siRNA treated macrophages after exposure to different concentrations of DCA. **(C)** ELISA of IL-1β (left) and analyses of LDH (right) in the supernatants of *hulncRNA57RIK* shRNA (sh57Rik) transfected macrophages after exposure to different stimulators. **(D)** ELISA of IL-1β (left) and analyses of LDH (right) in the supernatants of exogenous *hulncRNA57RIK* (oe57Rik) transfected macrophages after exposure to different stimulators. **(E)** Fluorescence *in situ* hybridization of *hulncRNA57RIK* and QRT-PCR of *hulncRNA57RIK* in the cytosol and nucleus of S1PR2 siRNA transfected human macrophages upon exposure to DCA for 12 hours. Nuclei were stained with DAPI (blue); Green, *hulncRNA57RIK*. Scale bar, 2.5 μM. NC, no treated control. **(F)** Northern blot of *hulncRNA57RIK* in S1PR2 siRNA transfected human macrophages upon exposure to DCA for 12 hours (left) and quantification of the blotting(right). **(G)** QRT-PCR of *hulnc57Rik* in Gα12/13, Gαi and Gαq siRNA treated macrophages after exposure to DCA. **(H)** QRT-PCR of *hulnc57Rik* in different signaling pathway inhibitors treated macrophages after exposure to DCA. CMC2.24, Ras inhibitor; SCH772984, ERKs inhibitor; Wortmannin, PI3K inhibitor; JNK-IN-8, JNK inhibitor; Perifosine, AKT inhibitor. **(I & J)** Analyses of H3K4me3 modification on the promoter region of *lncRNA57RIK* (G) and CHIP-PCR (H) for binding sites of H3K4me3 in the promoter region of *lncRNA57RIK* in S1PR2 siRNA or exogenous S1PR2 transfected macrophages after exposure to DCA. ChIP assays were performed using anti- H3K4me3 and normal rabbit IgG and then qRT-PCR.** (K)** CHIP-PCR for binding sites of H3K4me3 in the promoter region of *lncRNA57RIK* in DPY30, RBBP5 and WDR5 siRNA treated macrophages after exposure to DCA. **(L)** QRT-PCR of *hulnc57Rik* in DPY30, RBBP5 and WDR5 siRNA treated macrophages after exposure to DCA. **(M)** QRT-PCR (left) and fluorescence (right) *in situ* hybridization of *hulncRNA57RIK* in the macrophages of healthy individuals and patients. R. E, relative expression. Nuclei were stained with DAPI (blue); Green, *hulncRNA57RIK*. **(N)** Correlation analysis between *hulncRNA57RIK* in the monocytes/macrophages and bile acids in the sera of patients with cholestasis. **(O)** Correlation analysis between *hulncRNA57RIK* in the monocytes/macrophages and IL1β in the sera of patients with cholestasis. The relationship in (N and O) was analyzed using correlation and regression. R2, Pearson correlation coefficient. R. E, relative expression; ONE-way ANOVA Bonferroni's Multiple Comparison Test in (B); Mann-Whitney U test used in (M); Student's t-test in (C-L). **P < 0.01, ***P < 0.001, NS, not significant.

We next investigated how BA metabolite DCA promoted LPS-mediated production of IL-1β and pyroptosis. We first performed RNA-seq in the human macrophages upon exposure to BA metabolite DCA. A cluster of genes was upregulated (Figure 2A). Since DCA mediating mIL-1β production and pyroptosis is via S1PR2, we further analyzed which kind of gene (s) was modulated via S1PR2. Several S1PR2 associated genes were demonstrated by S1PR2 siRNA or exogenous S1PR2 transfected human macrophages (Figure S2A). We next determined potential function of these genes in controlling production of IL-1β. Data showed that human *lncRNA57RIK* (*hulncRNA57RIK*) was involved in LPS mediated production of mIL-1β and pyroptosis of human monocytes/macrophages (Figure 2B, C and Figure S2B-D). Overexpression of *hulncRNA57RIK* also confirmed the involvement of this lncRNA in LPS-mediated IL-1β maturation and pyroptosis of human macrophages (Figure 2D and Figure S2D). This *hulncRNA57RIK* belonged to intergenic *lncRNA*, which was predominately localized to the cytoplasm and did not encode a protein (Figure 2E and Figure S3A, B). The less expression of *hulncRNA57RIK* in S1PR2 shRNA transfected human macrophages upon exposure to BA metabolites was also further confirmed using Northern blot (Figure 2F). Since S1PR2 could activate multiple signal pathway such as that S1PR2 triggers the downstream RHO pathway and cAMP pathway via coupling the G_α12/13_ protein, that S1PR2 coupling G_αi_ protein induces PI3K, MAPK cascade reaction, and that S1PR2 coupling to G_αq_ protein triggers downstream phosphatidylinositol signaling pathway 51, we first used G_α12/13,_ G_αi_ and G_αq_ siRNA, and then using inhibitors CMC2.24 (Ras inhibitor), SCH772984 (ERKs inhibitor), Wortmannin (PI3K inhibitor), JNK-IN-8 (JNK inhibitor), and Perifosine(AKT inhibitor) to investigate DCA mediated signal pathway(s). Results showed that Gαi mediated PI3K and AKT signal pathways play a critical role in S1PR2 mediated expression *hulncRNA57RIK* (Figure 2G and H). In addition, the region of promoter on the *hulncRNA57RIK* also showed the less enrichment of H3K4me3 in S1PR2 shRNA transfected macrophages upon exposure to BA metabolites (Figure 2I, J), suggesting that BA metabolites mediated expression of *hulncRNA57RIK* was via the enrichment of H3K4me3, which can promote the expression of genes 52. DPY30, RBBP5 and WDR5 are best characterized as adaptor protein of the COMPASS complex that catalyze Histone3 lysine 4 di- and tri-methylation (H3K4me2,3) 53. We used DPY30, RBBP5 and WDR5 siRNAs to further investigate these. Data showed that H3K4me3 enrichment in promoter region of *lncRNA57RIK* indeed was important in the *lncRNA57RIK* expression (Figure 2K and 2L). All of these demonstrate that S1PR2 is involved in BA metabolites-mediated expression of *hulncRNA57RIK* in human macrophages*.* We also detected the *lncRNA57RIK* levels in the peripheral blood monocytes/macrophages of patients with cholestatic jaundice. *HulncRNA57RIK* was also much higher in the monocytes/macrophages of patients with cholestasis (Figure 2M). Interestingly, there existed positive relationship between the levels of BAs in the sera and the levels of* hulncRNA57RIK* in the monocytes/macrophages of patients with cholestasis (Figure 2N). The positive relationship between the levels of* hulncRNA57RIK* in the monocytes/macrophages and the levels of IL-1β in the sera of patients with cholestasis was also observed (Figure 2O). Taken together, we demonstrate that BA metabolite DCA can promote expression of *hulncRNA57RIK* via S1PR2, which might have a potential role in regulating the expression of IL-1β.

### *HulncRNA57RIK* mediated IL-1β maturation and pyroptosis is via the binding of caspase-4 with GBP1 in human macrophages

**Figure 3:**
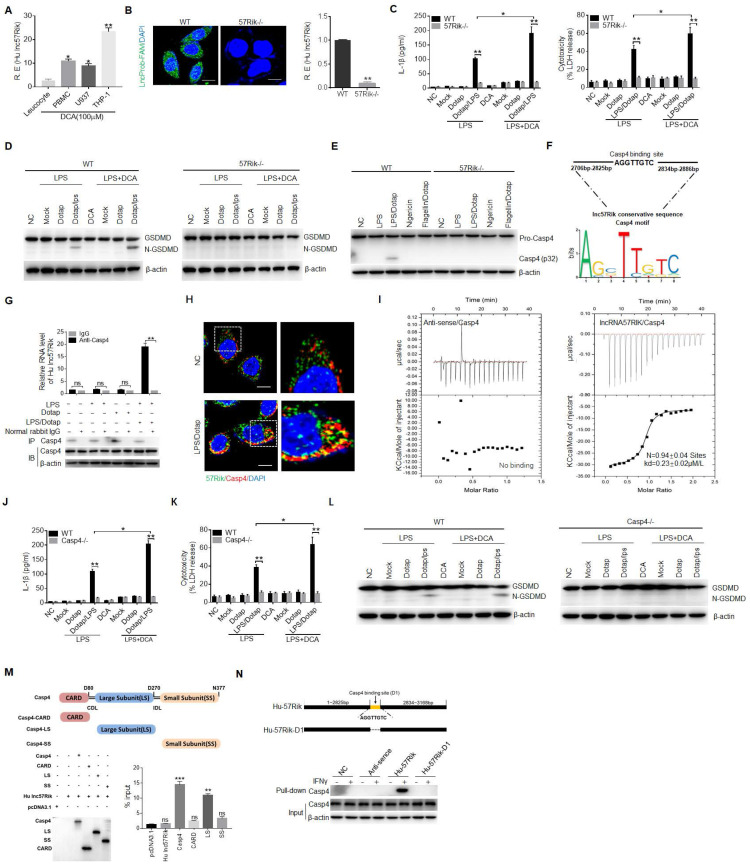
***HulncRNA57RIK* binds with caspase-4. (A)** QRT-PCR of *hulncRNA57RIK* in PBMCs, U937 and THP1 cells after exposure to DCA for 12 h. R. E, relative expression. **(B)** FISH (left) and qRT-PCR (right) of *hulncRNA57RIK* in THP1 cells. Green, *hulncRNA57RIK*; Blue, nuclei. Scale bar, 2.5 μM; 57Rik-/-, *hulncRNA57RIK*-/- macrophages. **(C)** ELISA of IL-1β (left) and analyses of LDH (right) in the supernatants of wt and 57Rik-/- THP1 cells upon exposure to different stimulators. Wt and 57Rik-/- THP1 cells were pretreated with LPS or LPS+DCA, then stimulated by LPS or LPS with Dotap. **(D)** Immunoblots of GSDMD in wt and 57Rik-/- THP1 cells upon exposure to different stimulators. **(E)** Immunoblots of caspase-4 in wt and 57Rik-/- THP1 cells upon exposure to different stimulators. Wt and 57Rik-/- THP1 cells were pretreated with LPS or LPS+DCA, then stimulated by nigericin, flagellin with dotap and LPS /Dotap. Cell lysates were assayed with caspase-4 antibody. **(F)** The putative caspase-4 binding site in the *hulncRNA57RIK* conservative sequence. Sequence logo of caspase-4 binding motif was obtained used the MEME software (https://meme-suite.org/meme/). **(G)** RIP of the THP1 cells upon exposure to Dotap or LPS with Dotap. Cell lysates were incubated with normal rabbit IgG and caspase-4 antibody. The immunoprecipitations were analyzed by QRT-PCR to examine enrichment efficiency of *hulncRNA57RIK*. **(H)** RNA-FISH of caspase-4 and *hulncRNA57RIK* in THP1 cells upon exposure to LPS with Dotap. Red, caspase-4; Green, *hulncRNA57RIK*; Blue, nuclei. Scale bar, 2.5 μM. **(I)** ITC analysis of the binding of *hulncRNA57RIK* with Caspase 4. **(J & K)** ELISA of IL-1β (J) and analyses of LDH (K) in the supernatants of wt and caspase-4 -/- THP1 cells upon exposure to different stimulators. THP1 cells were pretreated with LPS or LPS+DCA for 6 hours, then stimulated by Dotap or LPS with Dotap for 2h. **(L)** Immunoblots of GSDMD in wt and Caspase 4 -/- THP1 cells upon exposure to different stimulators. **(M)** RIP of V5-tagged caspase4 derivatives and *hulncRNA57RIK* cotransfected HEK293T cells. RIP was performed using anti-V5. % input of *hulncRNA57RIK* was analyzed. Caspase 4 and its derivatives were cloned into pcDNA3.1/V5 to generate V5-tagged- caspase4 and V5-tagged caspase4 derivatives, and then individually transfected into HEK293T cells. **(N)** RNA-pull down analyses using biotinylated *hulncRNA57RIK* and *hulncRNA57RIK* fragments in THP1 cells. *HulncRNA57RIK* plasmids which lack caspase-4 binding sites “D1” were constructed. NC, no biotinylated *hulncRNA57RIK* and fragments. ONE-way ANOVA Bonferroni's Multiple Comparison Test in (A and M); Student's t-test in (B, C, G, J and K); *P < 0.05, **P < 0.01, ***P < 0.001, NS, not significant.

Not only human monocytes/macrophages but also monocyte/macrophage cells line U937 and THP1 expressed *hulncRNA57RIK* (Figure 3A). To further determine function(s) of DCA mediated *hulncRNA57RIK* in the human macrophages, we generated *hulncRNA57RIK* knockout (KO) THP1 cells with demonstrated* hulncRNA57RIK* deficiency (Figure 3B). THP1, which can be induced into macrophages, is often used as a macrophage model of macrophages 54, 55. There were no changes in the IL-1β production and pyroptosis in *hulncRNA57RIK* KO THP1 cells with or without BA treatment upon exposure to caspase-4 ligand (Figure 3C); Whereas there were markedly differences in *hulncRNA57RIK* positive THP1 cells (Figure 3C), indicating that *hulncRNA57RIK* is involved in DCA mediated production of IL-1β and pyroptosis of monocytes/macrophages. Meanwhile, we also detected cleaved form of Gasdermin D. Data showed the fragments of Gasdermin D in wt but not* hulncRNA57RIK* KO cells (Figure 3D). Since caspase-4 ligands mediated production of IL-1β is though activation of caspase-4, we also examined whether caspase-4 could be activated in *lncRNA57RIK* KO macrophages upon exposure to caspase-4 ligand. No caspase-4 activation could be detected in the *lncRNA57RIK* KO macrophages (Figure 3E). These results demonstrate that *hulncRNA57RIK* is necessary for caspase-4 mediated production of mIL-1β.

Since *hulncRNA57RIK* is necessary for caspase-4-mediated production of mIL-1β, implying that *hulncRNA57RIK* is via caspase-4 to exert its role. LncRNAs could perform their function through encoding small peptides, interacting with microRNA, mRNA and proteins 56, 57. To exclude the effect of small peptides, we analyzed the coding potential of *lncRNA57RIK*. The phyloCSF showed that the unique exon of *lncRNA57RIK* did not have coding potential (Figure S3C, D). We next searched for open reading frames on the *lncRNA57RIK* sequence using the NCBI ORF finder software (https://www.ncbi.nlm.nih.gov/orffinder/) and found that there were 5 open reading frames (Figure S3E). However, Coding Potential Calculator 2 (CPC2, http://cpc2.gao-lab.org/) analyses revealed that these open reading frames did also not have the potential to encode peptides (Figure S3F). This was consistent with the results of *in vitro* overexpression experiments (Figure S3G), suggesting that *lncRNA57RIK* do not have the potential to encode small peptides. Next, we found that the levels of transcription and protein of caspase-4 did not significantly change between *lncRNA57RIK* KO macrophages and wild type (wt) human macrophages (Figure S4), suggesting that the role of *lncRNA57RIK* in the mIL-1β production and pyroptosis is not via regulating expression and translation of caspase-4. The interaction of some intracellular proteins with their substrates needs lncRNA 14-16. Bioinformatics analyses also predicted that this lncRNA could potentially bind with caspase-4 (Figure 3F and Tables S2 and S3). Indeed, RNA immunoprecipitation (RIP) showed the binding of *lncRNA57RIK* with caspase-4 (Figure 3G). Notably, this binding only happened upon exposure to caspase 4/11 ligands (Figure 3G). Immunofluorescence also confirmed the binding of *lncRNA57RIK* and caspase-4 (Figure 3H, I), suggesting that *lncRNA57RIK* is through the interaction with caspase-4 to exert its roles. Indeed, similar to *lncRNA57RIK* KO macrophages, reduced mIL-1β and pyroptosis could be found in the *caspase-4* KO macrophages upon exposure to caspase-4 ligands (Figure 3J, K). Data showed the fragments of Gasdermin D in wt but not in* caspase-4* KO cells to caspase-4 ligands (Figure 3L).

We also analyzed the domains of caspase-4, which potentially bind with *hulncRNA57RIK* (Figure 3M). Interaction of between the binding site (2825pb-2834pb) of *hulncRNA57RIK* and large subunit (LS) domain of caspase-4 was demonstrated by RIP and pulldown experiments (Figure 3N). Taken together, all of these indicate that *lncRNA57RIK* mediated IL-1β maturation and pyroptosis is via binding with caspase-4.

**Figure 4:**
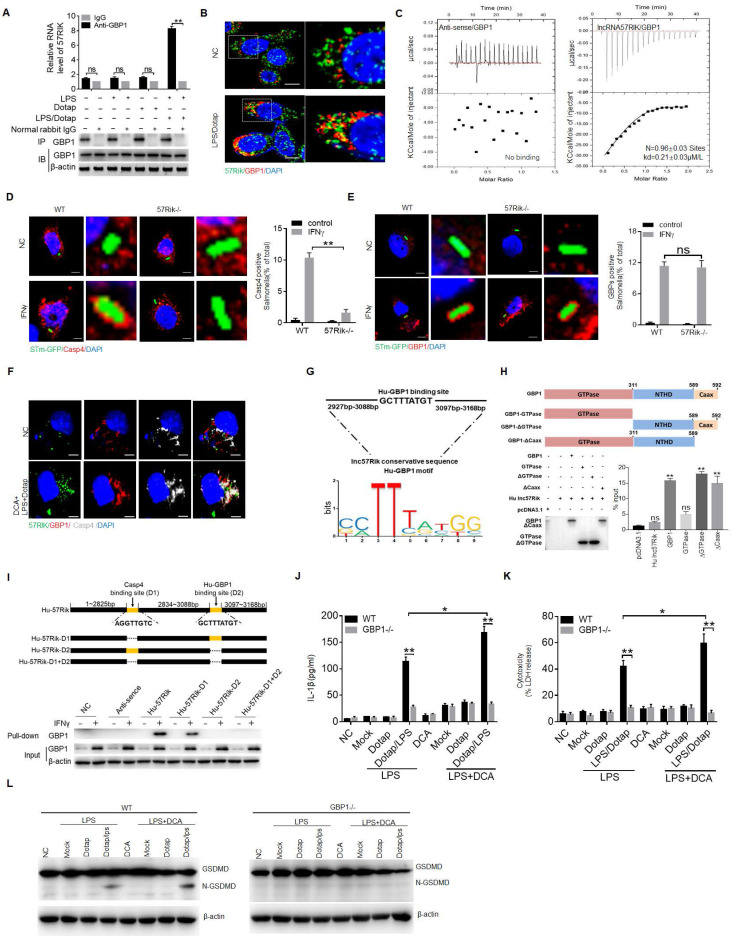
***HulncRNA57RIK* mediated IL-1β maturation is through promoting the binding of caspase-4 and GBP1. (A)** RIP of the THP1 cells upon exposure to Dotap or LPS with Dotap. Cell lysates were incubated with normal rabbit IgG and GBP1 antibody. Immunoprecipitations were analyzed by qRT-PCR to examine enrichment efficiency of *hulncRNA57RIK*. **(B)** Immunostaining and RNA-FISH of GBP1 and *hulncRNA57RIK* in the THP1 cells upon exposure to LPS with Dotap. Red, GBP1; Green, *hulncRNA57RIK*; Blue, nuclei. Scale bar, 2.5 μM. **(C)** ITC of the binding of *hulncRNA57RIK* with GBP1. **(D)** Fluorescence confocal microscopy in naive or IFNγ-primed wt and *hulncRNA57RIK* -/- THP1 cells, which were infected with GFP-labeled *Salmonella* for 1 h. Red, GBP1; Green, GFP-labeled *Salmonella*; Blue, nuclei. Scale bar, 2.5 μM.** (E)** Fluorescence confocal microscopy in naive or IFNγ-primed wt and *hulncRNA57RIK* -/- THP1 cells, which were infected with GFP-labeled *Salmonella* for 1 h. Red, caspase-4; Green, GFP-labeled Salmonella; Blue, nuclei. Scale bar, 2.5 μM. **(F)** Co-localization of lncRNA57RIK, GBP1, and CASP4 in macrophages. RNA-FISH of hulncRNA57RIK and immunostaining of Caspase 4/GBP1 in THP1 cells upon exposure to DCA+ (LPS+Dotap). NC, negative control. Red, GBP1; Green, hulncRNA57RIK; Grizzly, CAS4; Blue, nuclei. Scale bar, 2.5 μM. **(G)** The putative GBP1 binding site in the *hulncRNA57RIK* conservative sequence. **(H)** RIP of the V5-tagged GBP1 derivatives and *hulncRNA57RIK* co-transfected HEK293T cells. RIP was performed using anti-V5. % input of *hulncRNA57RIK* was analyzed. GBP1 and its derivatives were cloned into pcDNA3.1/V5 to generate V5-tagged- GBP1 and V5-tagged GBP1 derivatives, and then individually transfected into HEK293T cells. **(I)** RNA-pull down analyses using biotinylated *hulncRNA57RIK* and fragments in THP1 cells. GBP1 binding sites in *hulncRNA57RIK* were named as “D2”. Plasmids, which lacked *hulncRNA57RIK* D1, D2 sites and both D1 and D2 sites were constructed in pCDNA3.1. NC, empty plasmids. **(J & K)** ELISA of IL-1β (J) and analyses of LDH (K) in the supernatants of wt and GBP1 -/- THP1 cells upon exposure to different stimulators. THP1 cells were pretreated with LPS or LPS+DCA for 6 hours, then stimulated by Dotap or LPS with Dotap for 2h. **(L)** Immunoblots of GSDMD in wt and GBP1-/- THP1 cells upon exposure to different stimulators. ONE-way ANOVA Bonferroni's Multiple Comparison Test used in (H); Student's t-test in (A, D, E, J and K); *P < 0.05, **P < 0.01, NS, not significant.

Next, we looked for a mechanism for how *lncRNA57RIK* interacted with caspase-4 to causes more mIL-1β and pyroptosis of human monocytes/macrophages. LncRNAs could bind different proteins to exert their function 14-16. Since LPS mediated activation of caspase-4 is through the binding of caspase-4 with GBP1 9, it was possible for *lncRNA57RIK* to mediate the interaction of caspase-4 with GBP1. Thus, we next investigated whether *lncRNA57RIK* could also bind with CBP1. RIP and immunofluorescence staining showed the binding of *lncRNA57RIK* and GBP1 after exposure to caspase-4 ligands (Figure 4A-C). Data showed co-stained *lncRNA57RIK*, GBP1, and caspase 4 in macrophages after S1PR2 activation by DCA (Figure 4F). Notably, unlike to the *hulncRNA57RIK* positive cells, the binding of caspase-4 and GBP1 with intracellular *S.*T could not be detected in *hulncRNA57RIK* deficient cells upon exposure to IFNγ (Figure 4D, E), which can induce GBP1 expression 9. We also predicted the interacting sites of *hulncRNA57RIK* and analyzed the region of GBP1, which potentially binds with *hulncRNA57RIK* (Figure 4G, H and Tables S2 and S3). The interaction between the binding site (3088bp-3097bp) of *hulncRNA57RIK* and NTHD domain of GBP1 was also demonstrated by RIP and pull-down analyses (Figure 4I). Similar to *lncRNA57RIK* KO THP1 cells, reduced IL-1β maturation and pyroptosis could be found in *GBP1* KO THP1 cells (Figure 4J, K). Data showed the fragments of Gasdermin D in wt but not in *GBP1* KO THP1 cells (Figure 4L). Taken together, all of these suggest the* hulncRNA57RIK* mediated activation of caspase-4 by caspase-4/11 ligands is through the binding of *hulncRNA57RIK* with caspase-4 and GBP1 in human macrophages.

### *LncRNA57RIK* mediated IL-1β maturation and pyroptosis in the mouse macrophages is via a similar mechanism with human

**Figure 5:**
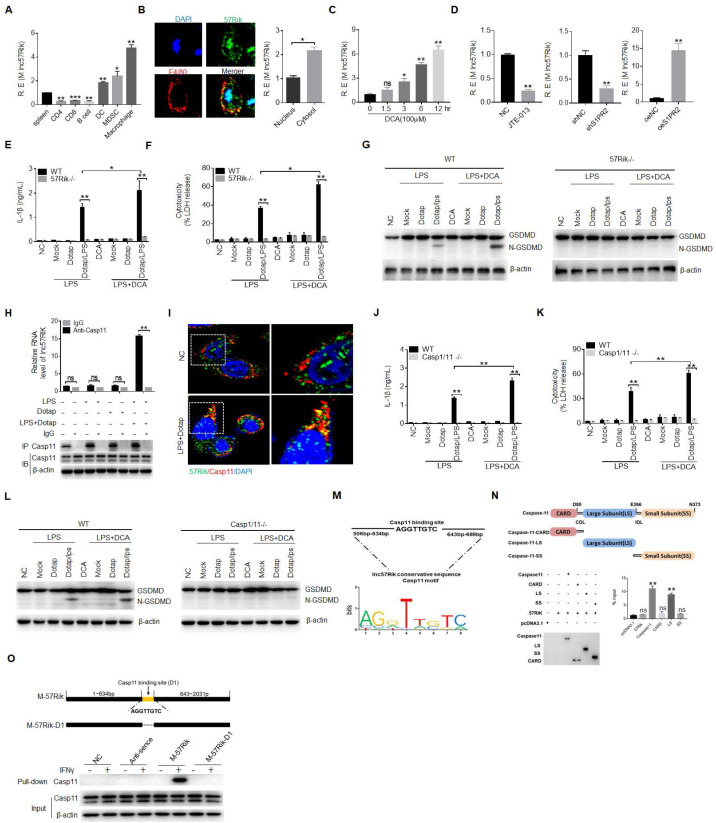
***MlncRNA57RIK* binds with caspase-11. (A)** QRT-PCR of *mlncRNA57RIK* in spleen, B cell, CD4, CD8, dendritic cells (DC), MDSCs and macrophages sorted from spleen by flow cytometry. R. E, relative expression. **(B)** FISH and qRT-PCR of *mlncRNA57RIK* in the cytosol and nuclei of mouse bone marrow derived macrophages (BMDMs). Nuclei were stained with DAPI (blue); Green, *mlncRNA57RIK*; Scale bar, 2.5 μM. **(C)** QRT-PCR of *mlncRNA57RIK* in BMDMs in different times upon exposure to DCA. R. E, relative expression. **(D)** QRT-PCR of *mlncRNA57RIK* in BMDMs treated with SIPR2 inhibitor (JTE-013, 10μM), siRNA (siS1PR2) and exogenous S1PR2 (oeS1PR2) after exposure to DCA. R. E, relative expression. **(E & F)** ELISA of IL-1β (E) and analyses of LDH (F) in the supernatants of the BMDMs of wt and *mlncRNA57RIK* -/- macrophages upon exposure to different stimulators. Macrophages were pretreated with LPS or LPS+DCA for 6 hours, then stimulated by Dotap or LPS with Dotap for 2h. **(G)** Immunoblots of GSDMD in wt and *mlncRNA57RIK* -/- macrophages upon exposure to different stimulators.** (H)** RIP of the BMDMs upon exposure to Dotap or LPS/Dotap. Cell lysates were incubated with normal rabbit IgG and caspase-11 antibody. The immunoprecipitations were analyzed by qRT-PCR to examine enrichment efficiency of *mlncRNA57RIK*. **(I)** RNA-FISH of caspase11 and *mlncRNA57RIK* in the BMDMs after exposed to LPS with Dotap. Red, caspase-11; Green, *mlncRNA57RIK*; Blue, nuclei. Scale bar, 2.5 μM. **(J & K)** ELISA of IL-1β (J) and analyses of LDH (K) in the supernatants of wt and *caspase-1/11* -/- BMDMs upon exposure to different stimulators. BMDMs were pretreated with LPS or LPS+DCA for 6 hours, then stimulated by Dotap (10μM) and LPS(2μg/mL) /Dotap for 2h. **(L)** Immunoblots of GSDMD in wt and caspase-1/11 -/- macrophages upon exposure to different stimulators. **(M)** The putative caspase-11 binding site in the *mlncRNA57RIK* conservative sequence. **(N)** RIP of the V5-tagged caspase-11 derivatives and *mlncRNA57RIK* cotransfected HEK293T cells. RIP was performed using anti-V5. % input of *mlncRNA57RIK* was analyzed. Caspase 11 and its derivatives were cloned into pcDNA3.1/V5 to generate V5-tagged- caspase 11 and V5-tagged caspase 11 derivatives, and then individually transfected into HEK293T cells. **(O)** RNA-pull down analyses using biotinylated *mlncRNA57RIK* and fragments in BMDMs. Caspase-11 binding sites were named as “D1”. The plasmids which lack *mlncRNA57RIK* “D1” sites were constructed. Nc, empty plasmids. ONE-way ANOVA Bonferroni's Multiple Comparison Test in (A, C and M); Two side Student's *t*-test in (B, C, G, J and K). *P < 0.05, **P < 0.01, ***P < 0.001, NS, not significant.

*LncRNA57RIK* was highly conserved between mouse and human with 52.12 % homology (https://blast.ncbi.nlm.nih.gov/Blast.cgi or DNAMAN software) (Figure S5A-C), Caspase-11 and caspase-4 also had a similar 3D structure (Figure S5D-F). We next investigated whether this *lncRNA57RIK* had also similar function in the human with mice. We first found that mouse* lncRNA57RIK (mlncRNA57RIK)* could be detected and regulated by BA metabolites in the mouse macrophages (Figure 5A-C). Data also showed that BA metabolites could promote the expression of *mlncRNA57RIK* through S1PR2 in the mouse macrophages (Figure 5D). Thus, we generated *mlncRNA57RIK* KO mice. There was no difference in the production of mIL-1β and pyroptosis in *mlncRNA57RIK* KO mouse macrophages with or without BA treatment upon exposure to caspase-11 ligands (Figure 5E, F); Whereas markedly differences could be detected in the macrophages from wt mice (Figure 5E, F), indicating that BA metabolites mediated *mlncRNA57RIK* plays a critical role in production of mIL-1β and pyroptosis of monocytes/macrophages. Data also showed the fragments of Gasdermin D in wt but not in* hulncRNA57RIK* KO macrophages to caspase-11 ligands (Figure 5G). All of these suggest that there exist similar functions in the *mlncRNA57RIK* of the macrophages between human and mice.

We next investigated the mechanism of mouse *mlncRNA57RIK* in promoting release of mIL-1β. Since *hulncRNA57RIK* in human could bind with caspase-4, we next investigated whether *mlncRNA57RIK* could also bind with caspase-11, which is homologous not only in structure but also in function with caspase-4 1-3. Results showed that *mlncRNA57RIK* could bind with caspase-11 after exposure to caspase-4/11 ligands (Figure 5H, I). Similar to *mlncRNA57RIK* KO macrophages, reduced production of mIL-1β and pyroptosis could also be found in the *caspase-1/11* KO mouse macrophages (Figure 5J, K). Data also showed the fragments of Gasdermin D in wt but not in caspase 1/11-/- macrophages (Figure 5L). We next analyzed potential interacting sites of *mlncRNA57RIK* and caspase-11 in mice (Figure 5M, N and Tables S2 and S3). RIP and pull-down analyses further confirmed the interaction between binding site (635bp-643pb) of *mlncRNA57RIK* and LS domain of caspase-11 (Figure 5O).

**Figure 6:**
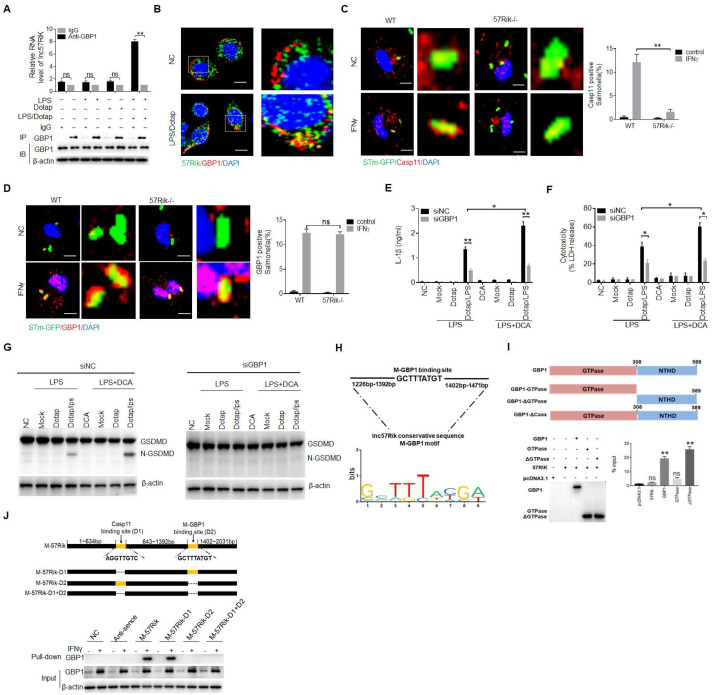
***MlncRNA57RIK* promotes the binding of caspase-11 with GBP1. (A)** RIP of the BMDMs upon exposure to Dotap or LPS with Dotap. Cell lysates were incubated with normal rabbit IgG and GBP1 antibody. **(B)** RNA-FISH of GBP1 and *mlncRNA57RIK* in the BMDMs after exposed to LPS with Dotap. Red, GBP1; Green, *mlncRNA57RIK*; Blue, nuclei. Scale bar, 2.5 μM. **(C)** Fluorescence confocal microscopy of naive or IFNγ-primed wt and *mlncRNA57RIK* -/- BMDMs, which were infected with GFP-labeled *Salmonella*. Red, caspase-11; Green, GFP-labeled *Salmonella*; Blue, nuclei; Scale bar, 2.5 μM. **(D)** Fluorescence confocal microscopy of naive or IFNγ-primed wt and *mlncRNA57RIK* -/- BMDMs, which were infected with GFP-labeled *Salmonella*. Red, GBP1; Green, GFP-labeled *Salmonella*; Blue, nuclei. NC, control transfection. Scale bar, 2.5 μM. **(E & F)** ELISA of IL-1β (F) and analyses of LDH (G) in the supernatants of wt, GBP1 siRNA (siGBP1) transfected BMDMs upon exposure to different stimulators. BMDMs were pretreated with LPS or LPS+DCA for 6 hours, then stimulated by Dotap or LPS with Dotap for 2h. NC, negative control. **(G)** Immunoblots of GSDMD in siNC and siGBP1 treated macrophages upon exposure to different stimulators. siNC, siRNA control. **(H)** The putative GBP1 binding site in the *mlncRNA57RIK* conservative sequence.** (I)** RIP of V5-tagged GBP1 derivatives and *mlncRNA57RIK* co-transfected HEK293T cells. RIP was performed using anti-V5. % input of *mlncRNA57RIK* was analyzed. GBP1 and its derivatives were cloned into pcDNA3.1/V5 to generate V5-tagged- GBP1 and V5-tagged GBP1 derivatives, and then individually transfected into HEK293T cells. **(J)** RNA-pull down analyses using biotinylated *mlncRNA57RIK.* GBP1 was analyzed in pull-down substance. GBP1 binding sites were named as “D2”. The plasmids which lack *mlncRNA57RIK* D1, D2 sites or both D1 and D2 sites were constructed. Nc, negative control. ONE-way ANOVA Bonferroni's Multiple Comparison Test in (I); Two side Student's *t*-test in (A, C, D, E and F). *P < 0.05, **P < 0.01, NS, not significant.

We next also determined whether *mlncRNA57RIK* also bound with GBP protein in mouse macrophages. Mouse GBP proteins include several proteins from GBP1, 2, 3, 5 to 7 5-8. However, there was absence of the report(s) on which kind of GBP to bind caspase-11. Thus, we used RIP to analyze these GBPs, which could potentially bind with *mlncRNA57RIK*. Data showed the binding of *mlncRNA57RIK* with GPB1 upon exposure to caspase-11 ligands (Figure 6A and Figure S6). The binding of *lncRNA57RIK* with GBP1 were further confirmed by immunofluorescence staining (Figure 6B). The *mlncRNA57RIK* deficiency also affected the binding of GBP1 and caspase-11 with intracellular *S.* T bacteria (Figure 6C, D). GBP1-silencing also reduced mIL-1β and pyroptosis with *mlncRNA57RIK* deficiency in the macrophages (Figure 6E, F). Data did not show the fragments of Gasdermin D in GBP1-silencing macrophages (Figure 6G). All of these support that *mlncRNA57RIK* to exert its role is through the binding of *mlncRNA57RIK* with caspase-11 and GBP1. We next also analyzed potential interacting sites in *mlncRNA57RIK* and the region of GBP1 of mice (Figure 6H, I and Tables S2 and S3). Interaction between binding site (1392bp-1402bp) of *mlncRNA57RIK* and NTHD domain of GBP1 was further confirmed through RIP and pull-down experiments (Figure 6J). Thus, similar to *hulncRNA57RIK*, *mlncRNA57RIK* can also mediate the interaction between caspase-11 and GBP1 to induce the activation of caspase-11 by LPS.

### DCA mediated *lncRNA57RIK* plays a critical role in LPS induced inflammation and pyroptosis in mice

**Figure 7:**
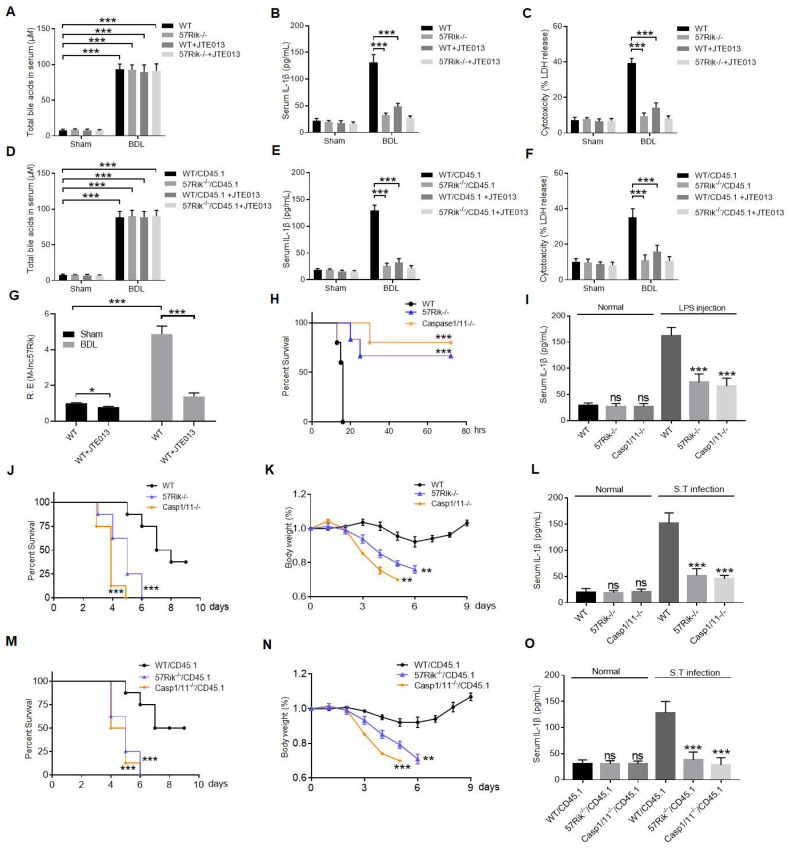
**DCA mediated *lncRNA57RIK* plays a critical role in LPS mediated inflammation. (A & B)** ELISA of total bile acids (A) and IL-1β (B) in the sera of wt and *mlncRNA57RIK* -/- mice with or without injecting JTE013 (30mg/kg) (n=6). JTE013, S1RP2 inhibitor; BDL, bile duct-ligated mice; Sham, mice without BDL. **(C)** Analyses of LDH in the supernatants of bone marrow cells (BMCs) of wt and *mlncRNA57RIK* -/- mice. BMCs from bile duct-ligated wt and *lncRNA57RIK* -/- mice were stimulated by LPS/Dotap. **(D & E)** ELISA of total bile acids (D) and IL-1β (E) in the sera of wt or *mlncRNA57RIK* -/- transplanted mice (n=6). Wt /CD45.1, BMCs from wt were transplanted into CD45.1 mice; 57Rik-/- / CD45.1, BMCs from *mlncRNA57RIK* -/- mice were transplanted into CD45.1 mice. **(F)** Analyses of LDH in the supernatants of the BMCs of wt and *mlncRNA57RIK* -/- transplanted mice. **(G)** QRT-PCR of *mlncRNA57RIK* in the BMCs of bile duct-ligated wt mice with or without injecting JTE013. BDL, bile duct-ligated mice. R. E, relative expression. **(H)** Survival rate of wt, *mlncRNA57RIK* -/- and *caspase-1/11* -/-mice with or without intraperitoneally injecting LPS (52mg/kg) (n=8). **(I)** ELISA of IL-1β in the sera of mice in (H). **(J & K)** Survival rate (J) and body weight (K) of wt, *lncRNA57RIK -/-* and *caspase-1/11 -/-* mice with or without *S.* T infection (200 CFUs/mouse, n=8). **(L)** ELISA of IL-1β in the sera from mice in (J and K). **(M & N)** Survival rate (M) and body weight (N) of wt, *mlncRNA57RIK* -/- and *caspase-1/11* -/- BMCs transplanted mice with or without *S.* T infection (200 CFUs/mouse, n=8). wt/CD45.1, BMCs from wt were transplanted into CD45.1 mice; 57Rik -/- / CD45.1, BMCs from *mlncRNA57RIK* -/- mice were transplanted into CD45.1 mice; Casp1/11 -/- /CD45.1, BMCs from *caspase1/11* -/- mice were transplanted into CD45.1 mice. **(O)** ELISA of IL-1β in the sera from mice in (M and N). ONE-way ANOVA Bonferroni's Multiple Comparison Test in (I, K, L, N and O); Wilcoxon's test in (H, J and M); Two side Student's *t*-test in (A-G). **P < 0.01, ***P < 0.001, NS, not significant.

We finally determined the role of macrophage *mlncRNA57RIK* in LPS or Gram-negative bacteria mediated diseases. First, we performed bile tube ligation (BTL) analyses 22 (Figure 7A), data showed increased IL-1β in the sera of wt mice but not in *lncRNA57RIK* KO mice (Figure 7B). Meanwhile, we also observed more pyroptotic cells in the BMCs of wt mice but not in *lncRNA57RIK* KO mice upon exposure to caspase-11 ligand (Figure 7C). We also performed BTL analyses in bone marrow-transplantation (BMT) mice to determine the roles of *lncRNA57RIK* in macrophages. Results showed that the increased IL-1β in the sera and pyroptosis of the monocytes/macrophases depended on the *lncRNA57RIK* in the macrophages upon exposure to caspase-11 ligands in wt transplanted *mlncRNA57RIK* -/- mice (Figure 7D-G). LPS toxic analyses showed that *mlncRNA57RIK* KO mice had higher survival rate than wt mice, similar to *caspase-11* KO mice (Figure 7H). These* lncRNA57RIK* KO mice in LPS toxic experiments had also decreased mIL-1β in sera (Figure 7I). To further determine the function of *mlncRNA57RIK* in the macrophages, we also employed *S.* T infection model. The *mlncRNA57RIK* KO, c*aspase-1/11* KO and wt mice were individually infused with *S.* T (200 CFUs/mouse). *LncRNA57RIK* KO mice had markedly reduced body weight, survival rate and serum levels of IL-1β (Figure 7J-L). Finally, we also performed BMT experiments in* S.* T infection model. Results showed that the resisting role of *lncRNA57RIK* in *S.* T infection models was dependent on *mlncRNA57RIK* in the BMCs (Figure 7M-O). Bacteria can colonize and infect anatomical sites other than the gastrointestinal tract of the host such as the lungs, liver and spleen 58. *LncRNA57RIK* KO mice also succumbed to infection more quickly and had increased bacterial burden in the lung, liver and spleen compared with wt mice (Figure S7). Taken together, all of these suggest that *mlncRNA57RIK* in the macrophages plays a critical role in LPS-mediated inflammation.

## Discussion

**Figure 8:**
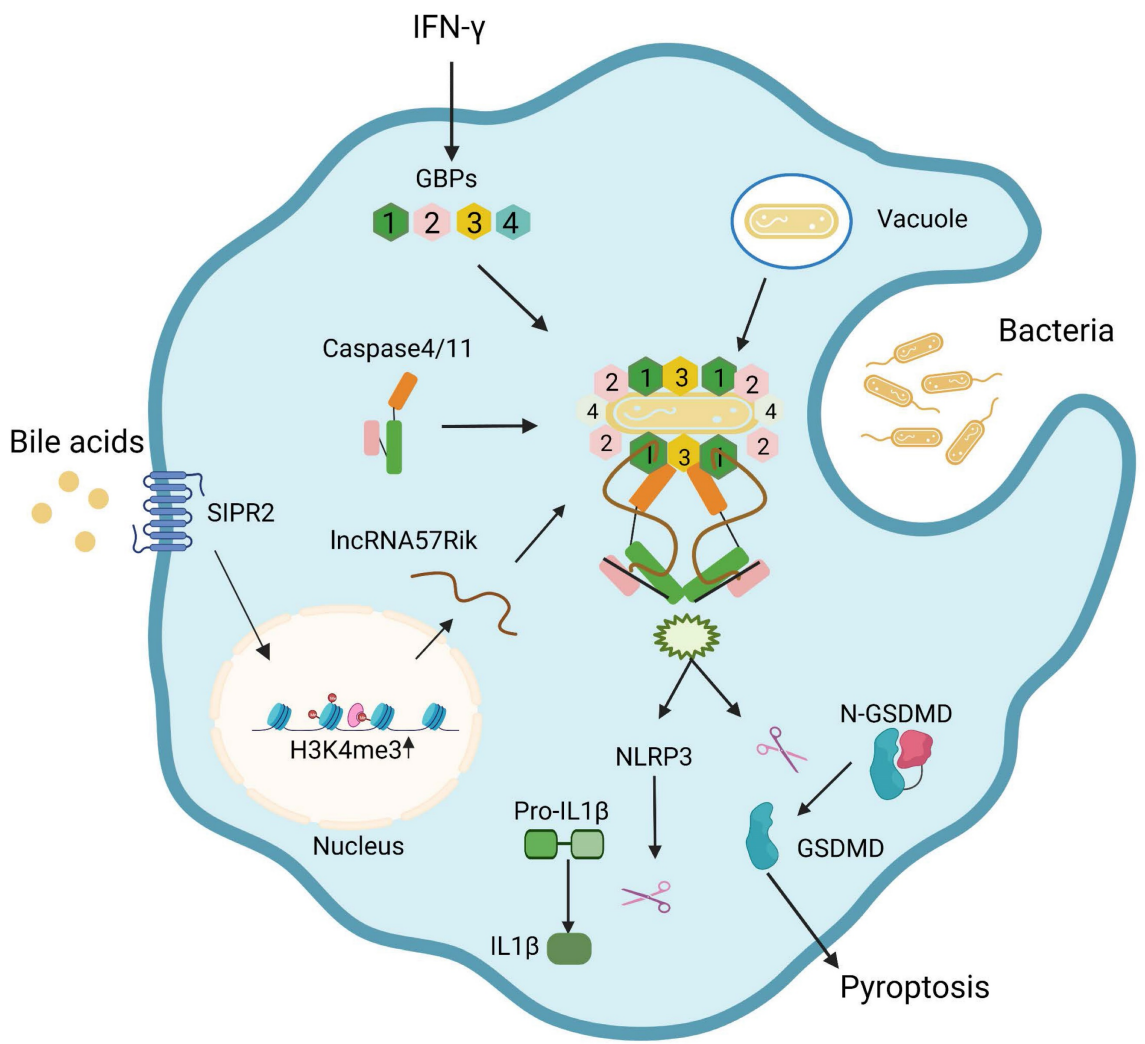
**Bile acid derivatives from gut microbiota promote GBPs-mediated activation of caspase-4/11 by LPS through *lncRNA57RIK*.** BA metabolites such as DCA induce *lncRNA57RIK* expression through SIPR2 mediated signal pathways, which can be promoted by H3K4me3 enrichments. The generated *LncRNA57RIK* can mediate the binding of caspase-4/11 with GBP1 induced by IFNγ to transfer LPS onto caspase-4/11, and finally activate caspase-4/11 to cause IL-1β maturation and cellular pyroptosis.

Gram-negative bacteria such as *Salmonella* can be bound by GBP1 and initiates the recruitment of GBP2-4 in the presence of bacterium LPS 9, 10, and then a complex formed by GBP-LPS promotes the recruitment of caspase-4/11 and subsequently transfers LPS onto caspase-4/11 to trigger its activation 9. It was not clear how GBP-LPS mediated the recruitment of caspase-4/11. We here found that BA metabolites generated by gut microbiota such as DCA play a critical role during this process. BA metabolite DCA can induce *lncRNA57RIK* expression. This lncRNA mediates the binding of caspase-4/11 with GBP1 to transfer LPS onto caspase-4/11, and finally activate caspase-4/11 to cause IL-1β maturation and cellular pyroptosis in the macrophages of mice and humans (Figure 8). Both murine *lncRNA57RIK* KO or human *hulncRNA57RIK* KO macrophages do not produce response to caspase-4/11 ligand or Gram-negative bacteria. These findings not only indicate the roles of BA metabolites-mediated *lncRNA57RIK* in the interaction of caspase-4/11 with GBPs but also explain why infection and sepsis are often found in the patients with cholestasis. Since BA metabolites can induce the expression of *lncRNA57RIK,* which determines the interaction of GBPs and caspase-4/11 upon exposure to LPS, this might also provide an explanation for excessive inflammation observed in patients with cholestasis.

We demonstrate that BA metabolites such as DCA can induce the expression of *lncRNA57RIK* via S1PR2 to cause inflammation and pyroptosis of the macrophages. Since previous reports indicated that BA receptors FXR and TGR5 deficient macrophages had reduced caspase-1/11 activation and release of mIL-1β upon *E. coli* infection 28, there seem to be contradict in the regulation of BAs on the regulations of macrophages. The different roles of BAs might depend on the different receptors mediated pathways 25. There have multiple receptors of BAs in the macrophages, including a range of nuclear and membrane receptors 59. We here found that BA metabolite DCA mediated IL-1β maturation and pyroptosis is through S1PR2, which can mediate expression of *lncRNA57RIK*. Other studies also found that S1PR2 signaling promoted caspase-11 dependent macrophage pyroptosis and worsened *E. coli* sepsis outcome 30. S1PR2 deficiency decreased macrophage pyroptosis and improved survival in *E. coli* sepsis 30 and also significantly reduced inflammation and liver fibrosis 60. Blockade of S1PR2 could also inhibit S1P-induced NLRP3 inflammasome priming and inflammatory cytokine (IL-1β and IL-18) secretion 31. S1PR2 has recently been reported as a conjugated BA-activated receptor 61. In addition, BA metabolite taurocholic acid (TCA) could also activate S1PR2 to promote immune cell infiltration and inflammation 60. Notably, it is hard to found BAs-free animal model (s) to determine whether the expression of *lncRNA57RIKs* only depends on BAs in the macrophages.

LPS from Gram-negative bacteria mediated caspase-11 (rodent) and caspases-4 (humans) (caspase-4/11) signaling appears in sepsis 62, diabetes 63, atherosclerosis 64, and Alzheimer's disease 65 in acute and chronic inflammatory conditions. A significant accumulation of BA levels was also often found in these diseases 66. Since *lncRNA57RIK* is necessary for the LPS from GBPs to activate caspase-4/11 to induce IL-1β maturation and pyroptosis of macrophages, BAs-mediated *lncRNA57RIK* should be important in the occurrence and development of these diseases. Thus, our data also offer a potential target for controlling these diseases, especially cholestasis -associated sepsis.

Gut microbiota derived metabolites not only impact the metabolism of the immune cells but also affect genetic and epigenetic regulation via their receptors in the immune cells 67-69. Our data exhibit that BA metabolite DCA from gut microbiota can promote the expression of *lncRNA57RIK*, an epigenetic factor to regulate the differentiation and function of macrophages.

## Supplementary Material

Supplementary figures and tables.
