# Supplementary material for: Bile acid derivatives from gut microbiota promote GBPs-mediated activation of caspase-4/11 by LPS through lncRNA57RIK
Source: Int J Biol Sci. 2024 Oct 28;20(15):5831–49. doi: 10.7150/ijbs.97059 (PMC11628326; doi:10.7150/ijbs.97059)
Supplement: Supplementary file 1 — Supplementary figures and tables. [file ijbsv20p5831s1.pdf]

Bile acid derivatives from gut microbiota promote GBPs-mediated activation of caspase-4/11 by LPS through *lncRNA57RIK*

Yunhuan Gao[1,2,3], Jianmei Yue[1,2,3], Fushuang Ha[4], Ya Wang[3], Rong Wang[3], Xiaorong Yang[3], Junqi Zhang[5], Xinqi Liu[5], Yuan Zhang[3], Tao Han[6] and Rongcun Yang [1,2,3]

Supplementary Figures

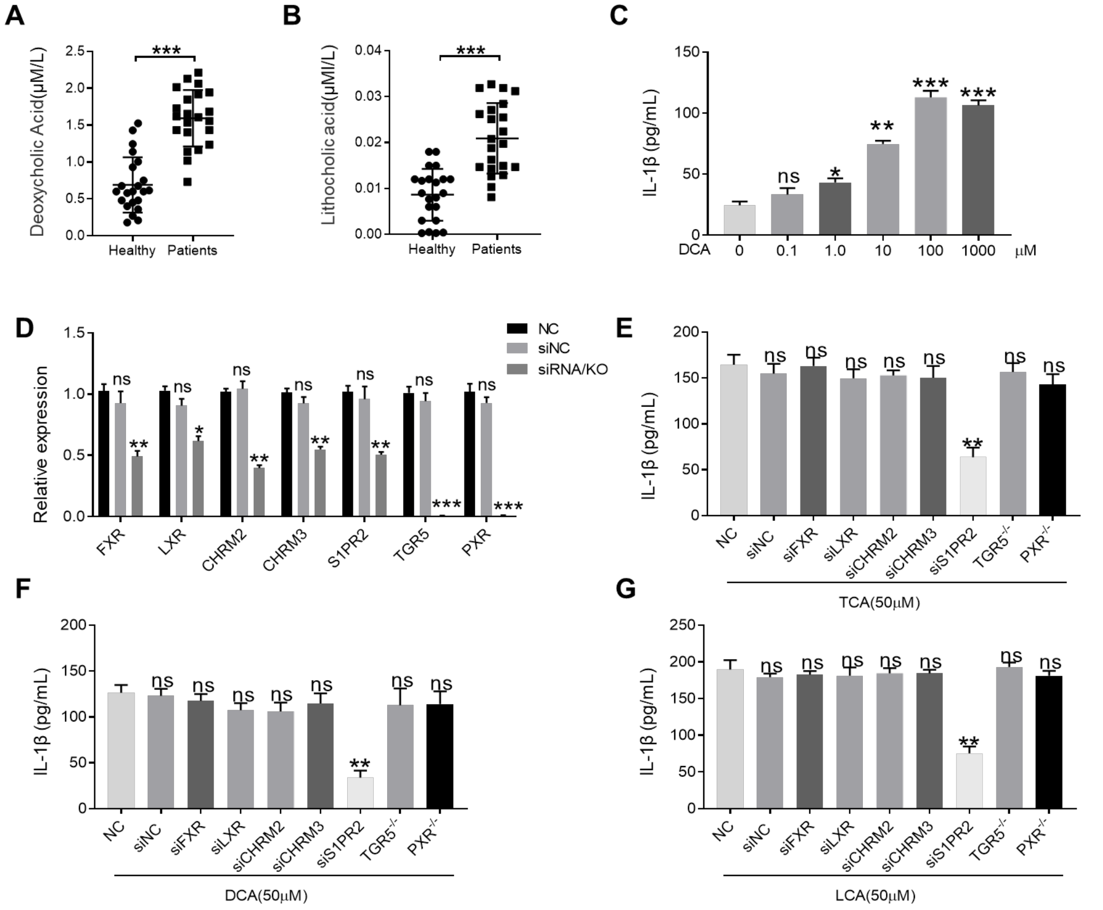

Figure S1. S1PR2 is involved in BAs mediated production of mIL-1β. (A) Analyses of deoxycholic acid (DCA) in the sera of healthy volunteers and patients with cholestasis using ELISA (n=22). **(B)** Analyses of Lithocholic acid (LCA) in the sera of healthy volunteers and patients with cholestasis using ELISA (n=22). **(C)** Effects of different concentration DCA on the IL-1 beta. The macrophages were exposed to different concentration of DCA 0.1, 1, 10, 100 and 1000 μM, and then IL-1β in the supernatants were detected using ELISA. **(D)** qRT-PCR of FXR, LXR, CHRM2, CHRM3, S1PR2 TGR5 and PXR in the macrophages after siRNA silencing FXR, LXR, CHRM2, CHRM3 and S1PR2 or knocking out TGR5 and PXR respectively. **(E)** ELISA of IL-1β in the supernatant of the macrophages after silencing FXR, LXR, CHRM2, CHRM3 or S1PR2 by siRNA or knocking out TGR5 and PXR respectively after exposure to TCA. **(F)** ELISA of IL-1β in the supernatant of the macrophages after silencing FXR, LXR, CHRM2, CHRM3 or S1PR2 by siRNA or knocking out TGR5 and PXR after exposure to DCA. **(G)** ELISA of IL-1β in the supernatant of the macrophages after silencing FXR, LXR, CHRM2, CHRM3 or S1PR2 by siRNA or knocking out TGR5 and PXR after exposure to LCA. The macrophages in (E, F and G) were pretreated with TCA, DCA or LCA respectively and stimulated by caspase 4 ligands (LPS with Dotap). A Mann–Whitney U test used

in (A and B), Student's t-test in (C-G). *P < 0.05, **P < 0.01, ***P < 0.001, NS, not significant.

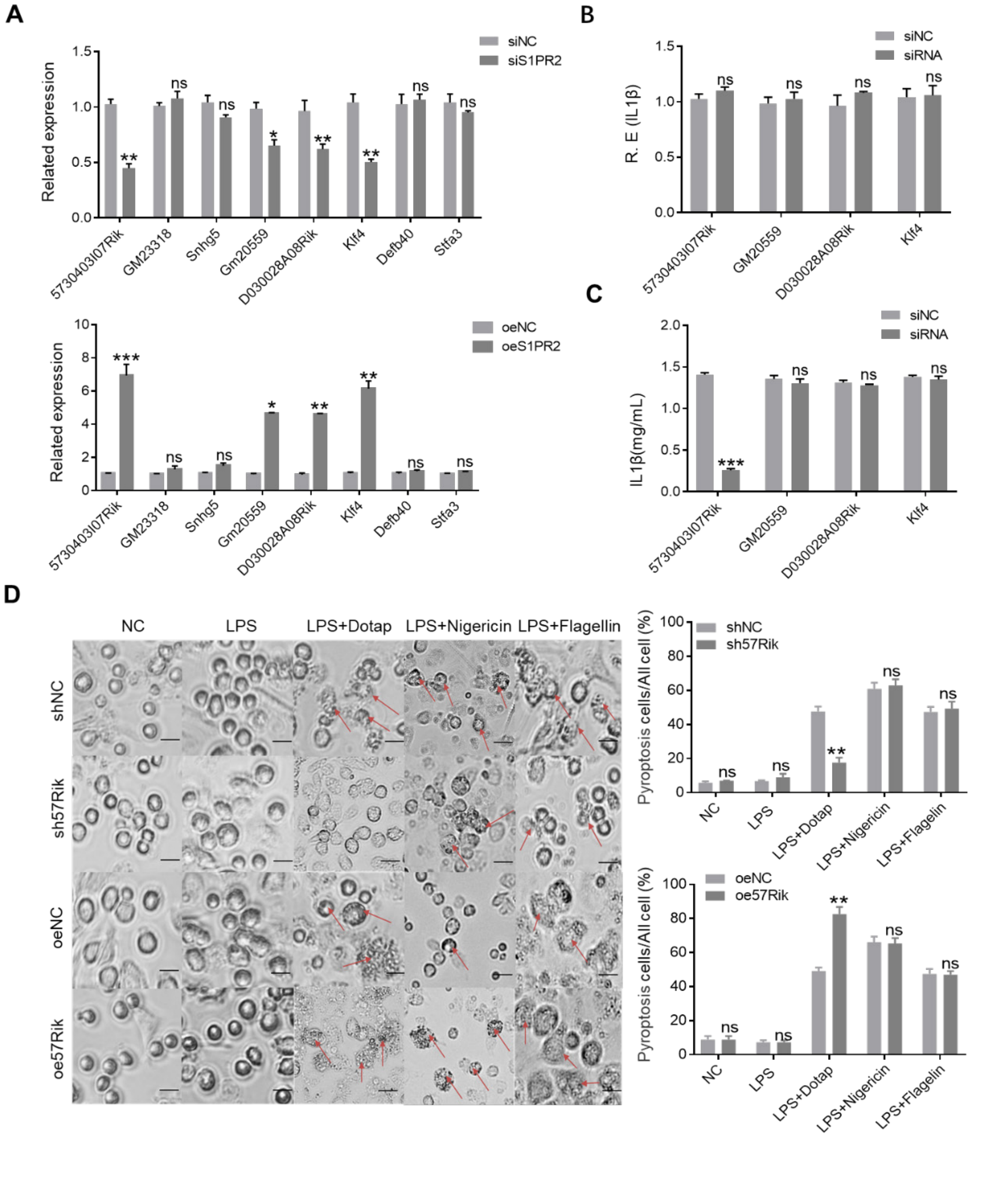

Figure S2. *LncRNA57RIK* is involved in BAs mediated production of mIL-1β. (A) qRT-PCR of the genes in the macrophages of wt mice, S1PR2 siRNA or exogenous S1PR2 transfected macrophages after exposure to DCA. **(B)** qRT-PCR of IL-1β in the macrophages after siRNA silencing S1PR2 relative genes. **(C)** ELISA of IL-1β in the supernatants of the macrophages after silencing S1PR2 relative genes. The treated macrophages were pretreated with DCA and stimulated by caspase 4 ligands (LPS with Dotap). **(D)** Pyroptotic cells and statistics of the lncRNA57RIK shRNA (sh57Rik) and exogenous lncRNA57RIK (oe57Rik) transfected macrophages under light microscopy after stimulated by LPS plus nigericin, LPS plus flagellin and LPS with

Dotap. Scale bar, 5 μM. The arrows indicate pyroptotic cells. Student's t-tests. *P < 0.05, **P < 0.01, ***P < 0.001, NS, not significant.

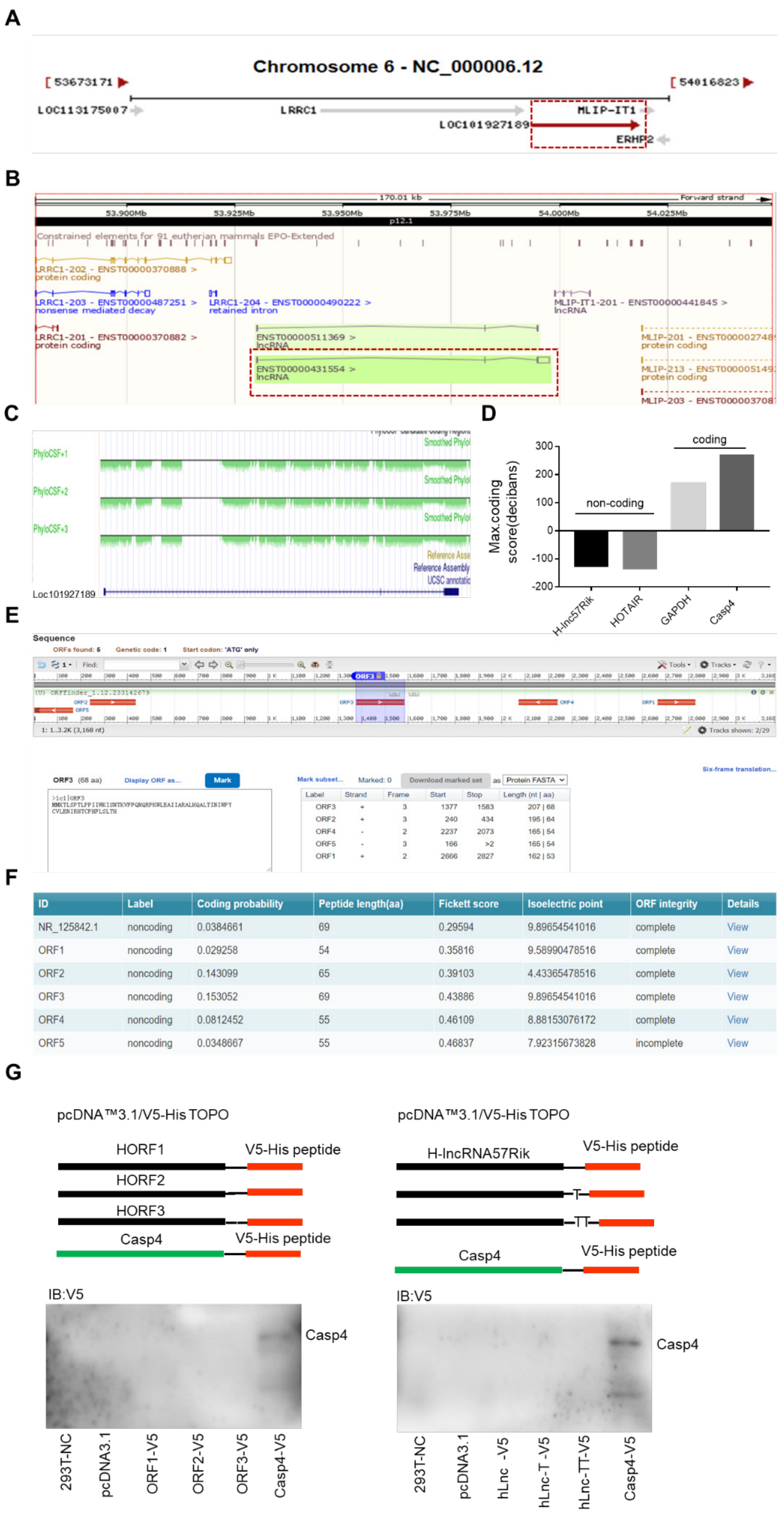

ID	Label	Coding probability	Peptide length(aa)	Fickett score	Isoelectric point	ORF integrity	Details
NR_125842.1	noncoding	0.0384661	69	0.29594	9.89654541016	complete	View
ORF1	noncoding	0.029258	54	0.35816	9.58990478516	complete	View
ORF2	noncoding	0.143099	65	0.39103	4.43365478516	complete	View
ORF3	noncoding	0.153052	69	0.43886	9.89654541016	complete	View
ORF4	noncoding	0.0812452	55	0.46109	8.88153076172	complete	View
ORF5	noncoding	0.0348667	55	0.46837	7.92315673828	incomplete	View

Figure S3. Characterization of *lncRNA57RIK*. (*A & B*) The location of lncRNA57

in chromosome. The NCBI database(https://www.ncbi.nlm.nih.gov/) and UCSC database(https://genome.ucsc.edu/) shows the chromosomal location of *HulncRNA57RIK*. **(*C* & *D*)** Coding potency of *HulncRNA57RIK* sequence was analyzed using PhyloCSF. Hotair, a control non-coding gene. GAPDH and caspase4, control coding genes. Scores above 0 suggested there had a coding potential, whereas scores below 0 represented no coding potential. **(E)** Open reading frame on *hulncRNA57RIK* by ORFfinder (https://www.ncbi.nlm.nih.gov/orffinder/). **(F)** Encoding capability of *hulncRNA57RIK* and open reading frame by CPC2 (Coding Potential Calculator2, http://cpc2.gao-lab.org/). **(G)** Immunoblotting of V5-tagged *HulncRNA57RIK* ORF transfected HEK293T cells. Different fragments of *hulncRNA57RIK* were cloned into pCDNA 3.1 plasmids and then transfected HEK293T cells. Immnoblotting was detected via anti-V5 antibody.

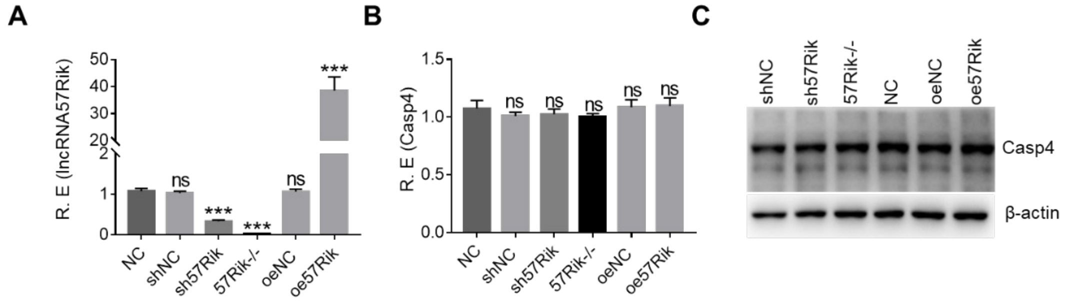

Figure S4. *LncRNA57RIK* does not affect the levels of caspase 4 mRNA and protein. (A) qRT-PCR of lncRNA57 in *lncRNA57* shRNA or exogenous *lncRNA57* transfected macrophages or *lncRNA57* KO macrophages. **(B)** QRT-PCR of caspase 4 in *lncRNA57* shRNA or exogenous *lncRNA57* transfected macrophages or *lncRNA57* KO macrophages. **(C)** Immunoblotting of caspase 4 in *lncRNA57* shRNA or exogenous *lncRNA57* transfected macrophages or *lncRNA57* KO macrophages. shNC, shRNA control; sh57Rik, *lncRNA57RIK* shRNA; oeNC, exogenous *lncRNA57RIK* control; oe57Rik, exogenous *lncRNA57RIK*; 57Rik-/-, *lncRNA57RIK* knockout. ONE-way ANOVA Bonferroni's Multiple Comparison Tests. ***P < 0.001, NS, not significant.

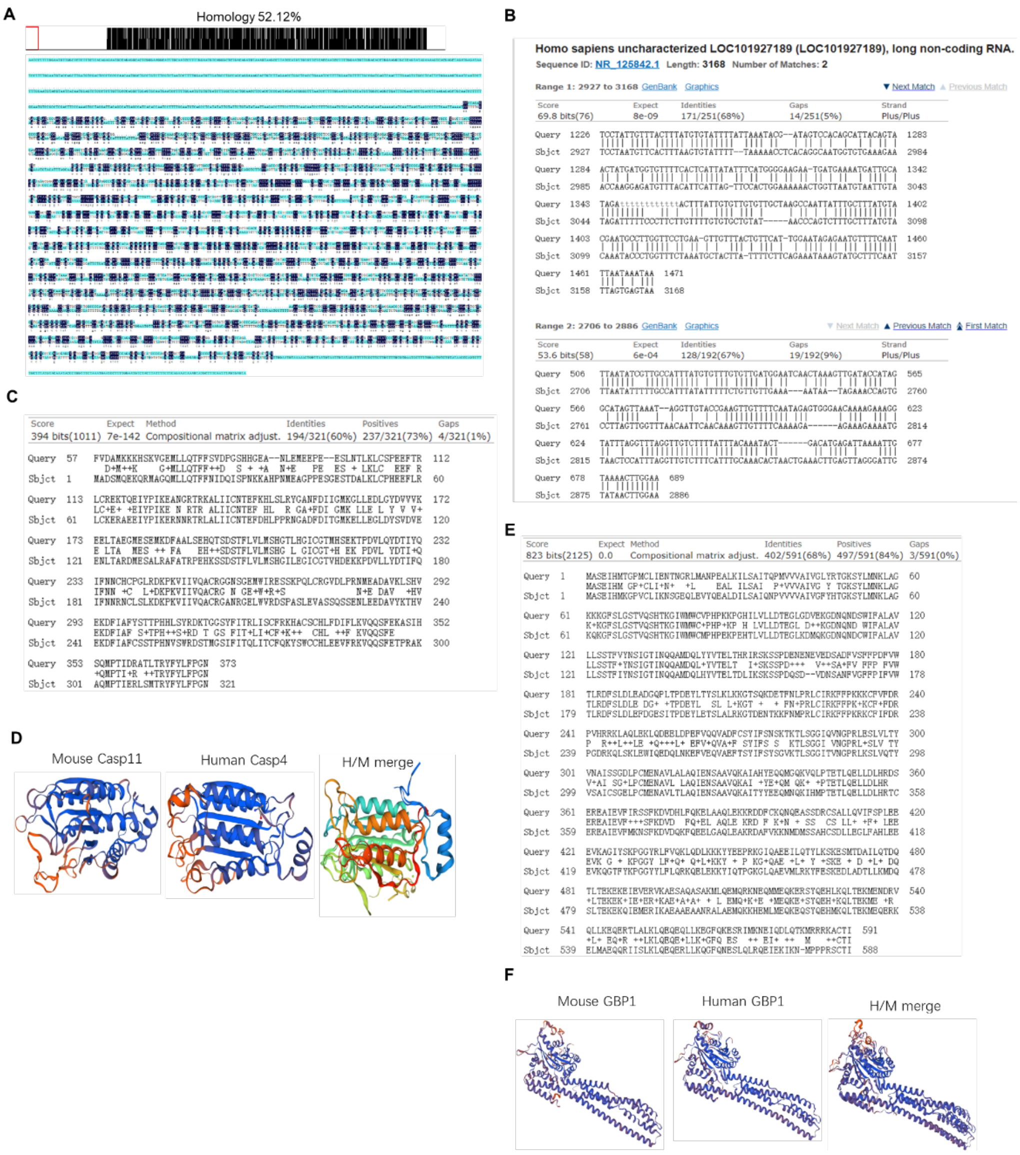

Figure S5. Higher conservation in *lncRNA57Rik*, caspase 4 and GBP1 between human and mice. (A & B) Comparison of lncRNA57Rik nucleic acid sequences in mice and humans use DNAMAN software (A) and NCBI database(https://blast.ncbi. nlm.nih.gov/Blast.cgi) (B). **(C)** Comparison of caspase4/11 nucleic acid sequences in mice and humans use NCBI database(https://blast.ncbi. nlm.nih.gov/Blast.cgi). **(D)** Crystal structure of mouse and human caspase 4/11 in the auto-inhibited conformation. We used SWISS-MODEL software to model the protein crystal structure of human caspase4/11 and compare the homology (https://swissmodel.expasy.org). **(E)** Comparison of GBP1 nucleic acid sequences in mice and humans use NCBI database (https://blast.ncbi.nlm. nih.gov/Blast.cgi). **(F)** Crystal structure of mouse and human GBP1 in the auto-inhibited conformation. We used SWISS-MODEL software to model the protein crystal structure of human GBP1 and compare the homology (https://swissmodel.expasy.org). H/M, human/mice.

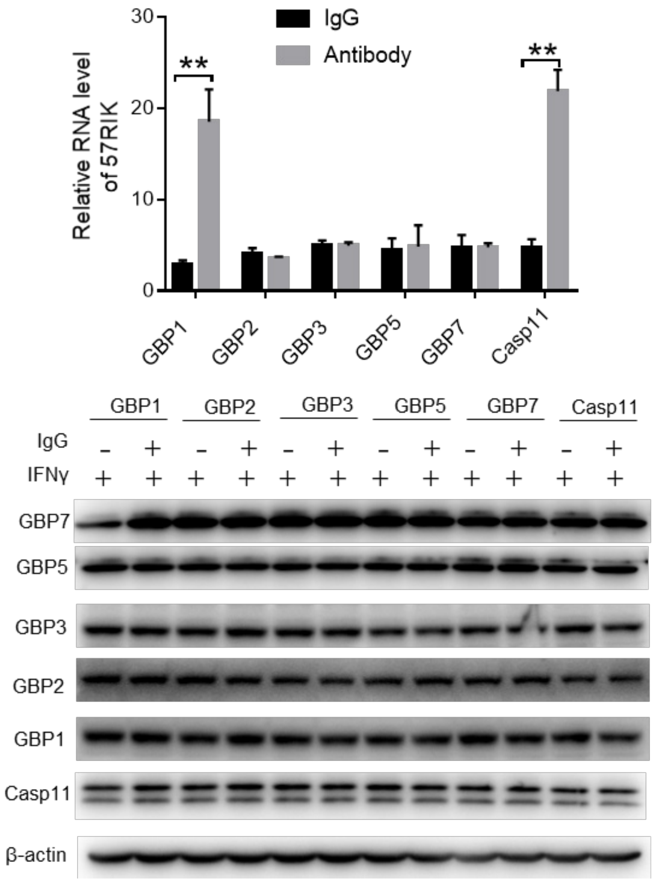

Figure S6. *MlncRNA57RIK* potentially binding with GBP1. RIP in the BMDMs after exposed to LPS/Dotap. Cell lysates were incubated with normal rabbit IgG and GBP-1/2/3/5/7 antibodies respectively. The immunoprecipitates were analyzed by QRT-PCR to exam enrichment efficiency of *mlncRNA57RIK*. Student's t-test; **P < 0.01, NS, not significant.

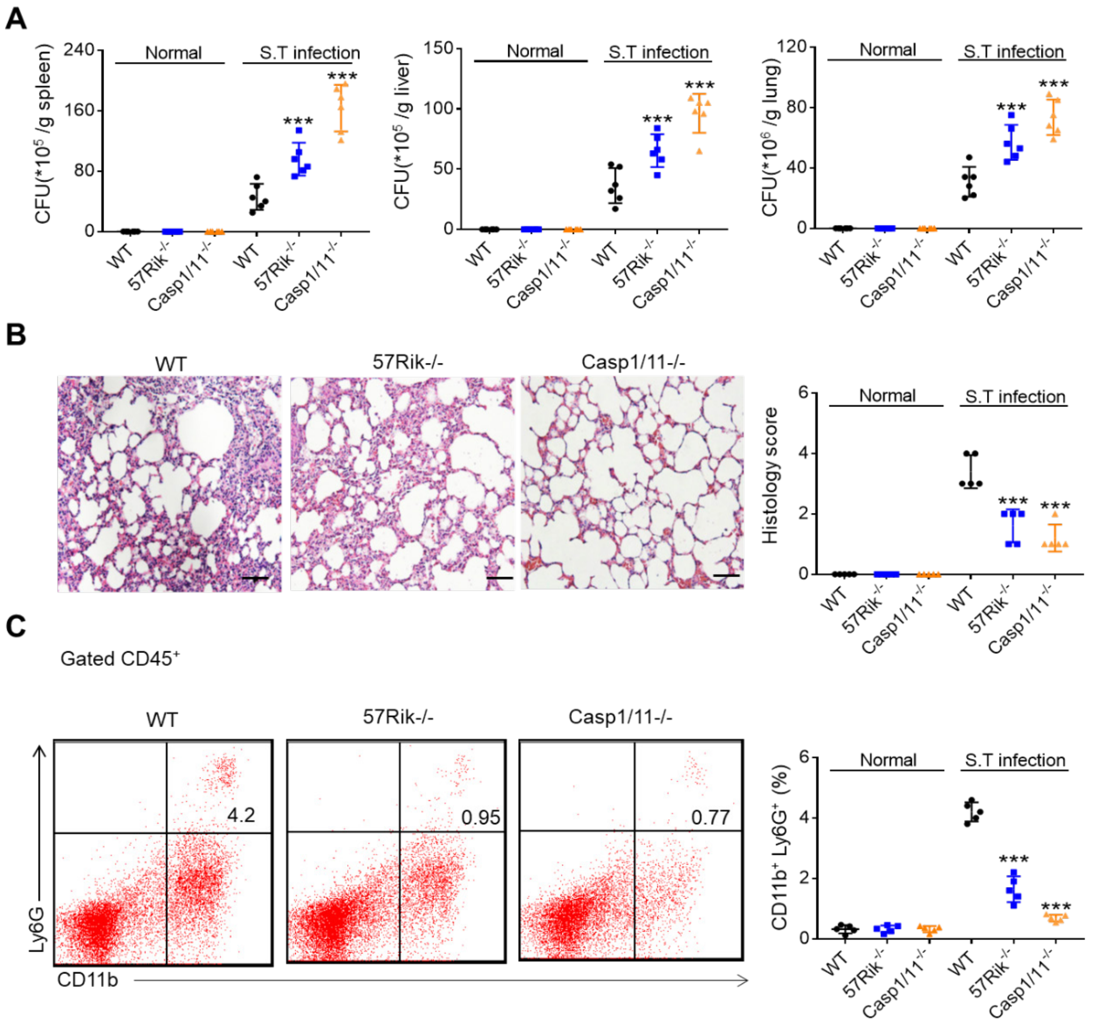

Figure S7. There are stronger inflammatory responses in WT mice than *lncRNA57RIK* KO or *caspase 1/11* KO mice after *S.*T infection. (A) CFUs of *S.* T bacteria in the spleen, liver and lung of wt, *lncRNA57RIK*-/-, and *caspase-1/11*-/- mice after *S.* T infection. Mice were from Figure7*J*. **(B)** H&E staining and histological score of lung sections from the mice. **(C)** Flow cytometry of neutrophils ($CD11b^{+}Ly6G^{+}$) from the intestinal lamina propria of mice in the Figure 7*J*. ONE-way ANOVA Bonferroni's Multiple Comparison Tests. ***$P < 0.001$, NS, not significant.

Table S1. Reagents and oligoes used in this study.

REAGENT or RESOURCE	SOURCE	IDENTIFIER
Antibodies		
β-Actin Antibody	Santa Cruz	Cat:sc-47778 RRID: AB_626632
Anti-Caspase 4 Antibody	Thermo Fisher Scientific	Cat: PA5-21286 RRID: AB_11153341
Anti-caspase-4 (4B9) Antibody	Santa Cruz Biotechnology	Cat: sc-56056, RRID: AB_781828
V5 Tag Monoclonal Antibody	Thermo Fisher Scientific	Cat:MA5-15253 RRID: AB_10977225
Anti-Caspase11 antibody	Cell Signaling Technology	Cat: 14340 RRID: AB_2728693
Anti-GBP1 antibody	Thermo Fisher Scientific	Cat: PA5-75381, RRID: AB_2719109
Anti-GBP2 antibody	Novus	Cat: NBP1-47774, RRID: AB_10010744
GBP3 Antibody	Affinity Biosciences	Cat: DF4114 RRID: AB_2836479
GBP5 Polyclonal antibody	Proteintech	Cat: 13220-1-AP RRID: AB_2109348
GBP7 Polyclonal Antibody	Thermo Fisher Scientific	Cat: PA5-24834 RRID: AB_2542334
Anti -IL1β antibody	Proteintech	Cat: 16806-1-AP RRID: AB_10646432
Anti-Histone H3 (tri methyl K4) antibody	Abcam	Cat: ab8580 RRID: AB_306649
Anti- Cleaved Caspase-1 antibody	Cell Signaling Technology	Cat：67314 RRID: AB_2714037
Anti-mouse GSDMD antibody	Abcam	Cat: ab209845 RRID: AB_2721254
Anti-human GSDMD antibody	Abcam	Cat: ab155233 RRID: AB_2736999
F4/80 (6A545) antibody	Santa Cruz	Cat:sc-71085 RRID: AB_1122717
APC-CD45 30-F11	Biolegend	Cat:103112 RRID: AB_312977
FITC-CD11b M1/70	eBioscience	Cat:11-0112-86 RRID: AB_464937
PE-Ly6G 1A8	Biolegend	Cat: 127607 RRID: AB_1186104
Bacterial and Virus Strains		
BL21 Chemically Competent cell	TransGen Biotech	Cat:CD901-01
DH5αChemically Competent cell	Tiangen Biotech	Cat:CD101-03
Human GAPDH cDNA clone	YouBio	Cat: G114877
S. Typhimurium	ATCC	Cat: 14028
Chemicals, Peptides, and Recombinant Proteins		
Recombinant Murine GM-CSF	PeproTech	Cat:315-03
Recombinant Murine M-CSF	PeproTech	Cat:315-02
Recombinant Human M-CSF	PeproTech	Cat: 300-25
Recombinant Human IFN-γ	PeproTech	Cat: 300-02

Recombinant Murine IFN-γ	PeproTech	Cat: 315-05
HiPerFect Transfection Reagent	QIAGEN	Cat:301705
Advanced DNA RNA Transfection Reagent	ZETA LIFE	Cat: AD600100
Chenodeoxycholic Acid (CDCA)	MCE	Cat: HY-76847
Taurocholic acid (TCA)	MCE	Cat: HY-B1788
Deoxycholic acid (DCA)	Selleck	Cat: S4689
Lithocholic acid (LCA)	MCE	Cat: HY-B0172
DOTAP chloride	Selleck	Cat: S6908
LPS (0111:B4)	Sigma	Cat: L2630
Nigericin	MedChemExpress	Cat: 28380-24-7
Flagellin	AdipoGen Life Sciences	Cat: AG-40B-0095
Phorbol 12-myristate 13-acetate (PMA)	selleck	Cat: S7791
Polybrene	Millipore	Cat:sc-134220
Lipofectamine™ 3000 Transfection Reagent	Thermo Fisher Scientific	Cat:11668027
Trizol	Life technologies	Cat:15596018
FXR antagonist 1(FXR inhibitor)	MCE	Cat: HY-151481
TEI-9648(VDR inhibitor)	MCE	Cat: HY-12398A
Larsucosterol (LXR inhibitor)	MCE	Cat: HY-139576
Resveratrol (PXR inhibitor)	MCE	Cat: HY-16561
SBI-115(TGR5 inhibitor)	MCE	Cat: HY-111534
CINPA1(CAR inhibitor)	MCE	Cat: HY-110249
JET-013(S1PR2 inhibitor)	Selleck	Cat: S7182
JNK-IN-8 (JNK inhibitor)	MCE	Cat: HY-13319
CMC2.24 (Ras inhibitor)	MCE	Cat: HY-120793
Wortmannin (PI3K inhibitor)	MCE	Cat: HY-10197
Perifosine (Akt inhibitor)	MCE	Cat: HY-50909
SCH772984 (ERKs inhibitor)	MCE	Cat: HY-50846
Experimental Models: Cell Lines		
HEK 293T	ATCC	N/A
THP-1	ATCC	N/A
U937	ATCC	N/A
Oligonucleotides for clone genes		
Murine lncRNA57Rik FW	BGI	5'-GTGACACAGGAGATCCCCAGA -3'
Murine lncRNA57Rik REV	BGI	5'-TCTGTTTCATTTATATTTATTGT-3'
Human ORF1 FW	BGI	5'- ATGATGAAAACATTGTCTCCT -3'
Human ORF1 REV	BGI	5'- TCAGTGAGTCAGGCTCAGAAA -3'
Human ORF2 FW	BGI	5'- ATGTCGACTCCCTCCCCAAC -3'
Human ORF2 REV	BGI	5'- TCACACCATTGCACTCCAGC -3'
Human ORF3 FW	BGI	5'- ATGCTTTTTACTCAGCTATG -3'
Human ORF3 REV	BGI	5'- CTAAATGGAGTTACATTTTC -3'

Human lncRNA57Rik FW	BGI	5'- TTGCTCTTGGTTTTAATCCTT -3'
Human lncRNA57Rik REV	BGI	5'-TTACTCACTAAATTGAAAGCAT-3'
Murine Caspase 11 FW	BGI	5'- ATGGCTGAAAACAAACACCC -3'
Murine Caspase 11 REV	BGI	5'- GTTGCCAGGAAAGAGGTAGA-3'
Human Caspase 4 FW	BGI	5'- ATGGCAGACTCTATGCAAGA-3'
Human Caspase 4 REV	BGI	5'- ATTGCCAGGAAAGAGGTAGAA-3'
Murine GBP1 FW	BGI	5'- ATGGCCTCAGAAATCCACAT-3'
Murine GBP1 REV	BGI	5'- AAGTATGGTGCATGATCGAG-3'
Human GBP1 FW	BGI	5'- ATGGCATCAGAGATCCACAT-3'
Human GBP1 REV	BGI	5'- GCTTATGGTACATGCCTTTC-3'
Human Caspase 4(CARD) FW	BGI	5'- ATGGCAGACTCTATGCAAGA -3'
Human Caspase 4(CARD) REV	BGI	5'- GCGTGTGCGGTTGTTTCTCT -3'
Human Caspase 4(LS) FW	BGI	5'-ATGCTGGCTCTCATCATATGCA-3'
Human Caspase 4(LS) REV	BGI	5'- GAGTTGTGTGATGAAGATAG -3'
Human Caspase 4(SS) FW	BGI	5'- ATGATCACATGCTTCCAGAAATA -3'
Human Caspase 4(SS) REV	BGI	5'- ATTGCCAGGAAAGAGGTAGAA -3'
Murine Caspase11 (CARD) FW	BGI	5'- ATGGCTGAAAACAAACACCC -3'
Murine Caspase11 (CARD) REV	BGI	5'- GTCCACACTGAAGAATGTCT -3'
Murine Caspase11(LS) FW	BGI	5'-ATGCCAGGCAGCCACCATGGT-3'
Murine Caspase11(LS) REV	BGI	5'- CTCTCTGATCCACATTTCTC-3'
Murine Caspase11(SS) FW	BGI	5'-ATGTCTTCAAAACCCCAGTTG-3'
Murine Caspase11(SS) REV	BGI	5'- GTTGCCAGGAAAGAGGTAGA-3'
Human GBP1(GTPase) FW	BGI	5'- ATGGCATCAGAGATCCACAT-3'
Human GBP1(GTPase) REV	BGI	5'- GCACGGCAGATCCCCACTGC-3'
Human GBP1(ΔGTPase) FW	BGI	5'- ATGGAGAACGCAGTCCTGGC-3'
Human GBP1(ΔGTPase) REV	BGI	5'- GCTTATGGTACATGCCTTTC-3'
Human GBP1 (ΔCaax) FW	BGI	5'- ATGGCATCAGAGATCCACAT-3'
Human GBP1(ΔCaax) REV	BGI	5'- ACATGCCTTTCGTCGTCTCA-3'
Human S1PR2 FW	BGI	5'-ATGGGCAGCTTGTACTCGGAG-3'
Human S1PR2 REV	BGI	5'- GACCACCGTGTTGCCCTCCAGA-3'
Murine GBP1(GTPase) FW	BGI	5'- ATGGCCTCAGAAATCCACAT -3'
Murine GBP1(GTPase) REV	BGI	5'- GGGTAGTTCTCCACTGCAGAT -3'
Murine GBP1(ΔGTPase) FW	BGI	5'- ATGTGTATGGAGAACGCAGTC -3'
Murine GBP1(ΔGTPase) REV	BGI	5'- AAGTATGGTGCATGATCGAG -3'
Oligonucleotides for qRT-PCR		
Murine GAPDH FW	BGI	5'-TCAACGGCACAGTCAAGG-3'
Murine GAPDH REV	BGI	5'-TACTCAGCACCGGCCTCA-3'
Murine lncRNA57Rik FW	BGI	5'- ATGTCTTTCCCCACCCTTCT -3'
Murine lncRNA57Rik REV	BGI	5'- ACCTTTCTTTTGTTCCCACT -3'
Murine Caspase11 FW	BGI	5'- GAGAAATGTGGATCAGAGAG-3'
Murine Caspase11 REV	BGI	5'- GATGTGGGGTTGTAGAGTAG-3'
Murine IL1β FW	BGI	5'-TCGCAGCAGCACATCAACAAG-3'
Murine IL1β REV	BGI	5'-GAAGGTCCACGGGAAAGACAC-3'
Murine GBP1 FW	BGI	5'- ATAGGAACCATCAACCAGCA-3'

Murine GBP1 REV	BGI	5'- ATCCCTCAGAGTCCACACAA-3'
Human GAPDH FW	BGI	5'- TCAAGAAGGTGGTGAAGCAGG-3'
Human GAPDH REV	BGI	5'- AGCGTCAAAGGTGGAGGAGTG-3'
Human lncRNA57Rik FW	BGI	5'- GCCTGTCTTCGTCTTGATGC -3'
Human lncRNA57Rik REV	BGI	5'- TACTGGGAGGTGGAGGTTGC -3'
Human Caspase4 FW	BGI	5'- AGAGGTGCAAACCGTGGGGAA -3'
Human Caspase4 REV	BGI	5'- CGTTGTGTGGCGTTGAAGAGC -3'
Human IL1β FW	BGI	5'- GGCAATGAGGATGACTTGTTC-3'
Human IL1β REV	BGI	5'- TGCTGTAGTGGTGGTCGGAGA-3'
Human GBP1 FW	BGI	5'- TCTGGATGTGGTGTGTGCCC -3'
Human GBP1 REV	BGI	5'- AGCCTGCTGGTTGATGGTTC -3'
Human FXR FW	BGI	5'- GCTGTGTGTTGTTTGTGGAG -3'
Human FXR REV	BGI	5'- GCGTTTTTGGTAATGCTTCT -3'
Human VDR FW	BGI	5'- CATCCCATTGCCCTGGTTATAT -3'
Human VDR REV	BGI	5'- TGTTTTTGTCTGTTTTTCCTCC -3'
Human CAR FW	BGI	5'- AATACCACTTTCTGTCTCCAAA -3'
Human CAR REV	BGI	5'- AGCTGATCAATCTCATCTCTCT -3'
Human S1PR2 FW	BGI	5'- TGCCTGTCCCGTCCACTCCT -3'
Human S1PR2 REV	BGI	5'- TCCGTCCTTGCACCCCCACC -3'
Human LXRa FW	BGI	5'-TCTGGAGACATCTCGGAGGT-3'
Human LXRa REV	BGI	5'-GATAGCAATGAGCAAGGCAA-3'
Human CHRM2 FW	BGI	5'-TCCATTAAAGTCAACCGCCA-3'
Human CHRM2 REV	BGI	5'-TCACACACCACAGGTCCCAA-3'
Human CHRM3 FW	BGI	5'- CTAGAGTCAGCCGTGGACAC -3'
Human CHRM3 REV	BGI	5'- TGATGAAGGCAAGCAAGATC-3'
GM23318 FW	BGI	5'- TCGCTTCGGTAGCAAATATACT -3'
GM23318 REV	BGI	5'- TTATTTAGTCCCCCCTCCCAG -3'
Snhg5 FW	BGI	5'- TTACGACGGAGCCTAAGATAT -3'
Snhg5 REV	BGI	5'- TTAGTACGAATCTCACTGGGGC -3'
GM20559 FW	BGI	5'- CTATGGCCTATCAAGTCAATAT -3'
GM20559 REV	BGI	5'- TAATTCGATAGACCTGTACTT -3'
D030028A08Rik FW	BGI	5'- GTATGGCTGTGAAGTGGC -3'
D030028A08Rik REV	BGI	5'- AGTTGTCCTGGGTAAAGG -3'
Klf4 FW	BGI	5'- CACCTCCCACGGCCCCCTTCA -3'
Klf4 REV	BGI	5'- TCAGAGACGCCTTCAGCACAA -3'
Defb40 FW	BGI	5'- TCCTGCTTTCTACTGATGATCT -3'
Defb40 REV	BGI	5'- TTTCTGAATGTGACAGTTGTTG -3'
Stfa3 FW	BGI	5'- AGATAGATGTAGGGAATGGTT -3'
Stfa3 REV	BGI	5'- AGTCTTGGTTTTGTTAGTCTG -3'
Human GNA12(Gα12) FW	BGI	5'- AGACCGTGAGCATCAAGAAGC -3'
Human GNA12(Gα12) REV	BGI	5'- GGTGAAGTGGTGGAAGAGTGG -3'
Human GNA13(Gα13) FW	BGI	5'- GGAGAAGGTGCAAATTGTGAG -3'
Human GNA13(Gα13) REV	BGI	5'- TGAAGTGGTGGTATAAGGGCT -3'
Human GNAQ(Gαq) FW	BGI	5'- TCCTCGGTTATTCTGTTCTTA -3'

Human GNAQ(Gαq) REV	BGI	5'- AATTTTGTCACTGTCTGGGTT -3'
Human GNAI1(Gαi1) FW	BGI	5'- CCCTCTCACTATATGCTATCC -3'
Human GNAI1(Gαi1) REV	BGI	5'- TATTTCCTTTGTGTCCTTTCT -3'
Human GNAI2(Gαi2) FW	BGI	5'- AGGGAATACCAGCTCAACGAC -3'
Human GNAI2(Gαi2) REV	BGI	5'- CTGACCACCCACATCAAACAT -3'
Human GNAI3(Gαi3) FW	BGI	5'- GGAGTGATTAAACGGTTATGG -3'
Human GNAI3(Gαi3) REV	BGI	5'- TGGAATGTAGTTAGACTGGGA -3'
Human WDR5 FW	BGI	5'- GAAGTGGATTGTGTCTGGCT -3'
Human WDR5 REV	BGI	5'- GATGATGTTTTCTGTTGGGT -3'
Human DPY30 FW	BGI	5'- TCCCTTTCCCCCACTTTCTC -3'
Human DPY30 REV	BGI	5'- GCATCTGCTCTGGCTCCATG -3'
Human RBBP5 FW	BGI	5'- GATGACTCCGATTTGAACGT -3'
Human RBBP5 REV	BGI	5'- ATGGCTGTGGTATTGCTTGT -3'
Oligonucleotides used in Northern blot		
T7- Human lncRNA57Rik -F	BGI	5'- CAAGACTGAGGTGACTCTGAG-3'
T7- Human lncRNA57Rik -R	BGI	5'-TAATACGACTCACTATAGGGCAGCACACAAAACAAGAAGGAAAA -3'
T7-U6 RNA-F	BGI	5'-GTGCTCGCTTCGGCAGCACATATAC-3'
T7-U6 RNA-R	BGI	5'-TAATACGACTCACTATAGGGAAAAATATGGAACGCTTCACGAATT-3'
Probes used in the RNA-FISH		
Murine lncRNA57Rik -FAM	BGI	5'-FAM-AATACCTTTCTTTTGTTCCCACTCT -3'
Human lncRNA57Rik -FAM	BGI	5'-FAM-CAGATAATAGGAGGCAGTGTAGGAG -3'
NC-FAM	BGI	5'-FAM-CGGGAGCCTAGGAAGTGCATCTTTC-3'
crRNAs used in this study		
Caspase 4	BGI	5'-AGGGATTCCAACACCTTAAG-3'
GBP1	BGI	5'-GAACACTAATGGGCGACTGA -3'
siRNA/shRNAs used in this study		
Murine lncRNA57Rik	Ribobio	5'-CCAGTCAGTGAAGCCTGCAATCACA-3'
Human lncRNA57Rik	Ribobio	5'-CAGGAGCGTTGCCATTGATGGCTAT-3'
Human FXR	Ribobio	5'-GACAGAGCCTCTGGATACCACTATA-3'
Human VDR	Ribobio	5'- CCACTGGCTTTCACTTCAATGCTAT-3'
Human CAR	Ribobio	5'-GACCAAGCCACAGGCTACCACTTTA-3'
Human S1PR2	Ribobio	5'-TGGCCATTGCCAAGGTCAAGCTGTA-3'

Human LXRa	Ribobio	5'-GGGCCATGAATGAGCTGCAACTCAA-3'
Human CHRM2	Ribobio	5'-CCTCAGTTATGAATCTGCTCATCAT-3'
Human CHRM3	Ribobio	5'-CCCGTGTGCTATGCTCTGTGCAACA-3'
Murine GBP1	Ribobio	5'-ACTGGACATCCTGTCTGCTATCCAA-3'
Gm20559	Ribobio	5'-CCCATCTTTGCATACACCAATGTTT -3'
D030028A08Rik	Ribobio	5'-GGGAATGGAGCAGTTTGACTCCCAT -3'
Klf4	Ribobio	5'-AGCAGCCCTTCGGTCATCAGTGTTA-3'
Human GNA12(Gα12)	Ribobio	5'-GCGACACCATCTTCGACAACA-3'
Human GNA13(Gα13)	Ribobio	5'-GGUGGUCAGAGAUCAGAAAGG-3'
Human GNAQ(Gαq)	Ribobio	5'-CGGUUAUUCUGUUCUUAAACA-3'
Human GNAI1(Gαi1)	Ribobio	5'-GGUUCUAGCUGAAGAUGAAGA-3'
Human GNAI2(Gαi2)	Ribobio	5'-GGGCGGUUGUCUACAGCAACA-3'
Human GNAI3(Gαi3)	Ribobio	5'-GCAAGAUGAUCGACCGCAACU-3'
Human WDR5	Ribobio	5'-GAAAGAGAUUGUACAGAAACU-3'
Human DPY30	Ribobio	5'-AGUUGCUACUGUAGAUUUACA-3'
Human RBBP5	Ribobio	5'-GGGCUAGUUCAGAGAAGAAGA -3'
Oligonucleotides used in CHIP-PCR		
P1 FW	BGI	5'-CATAGCAAGAAACCAGGCTTGT-3'
P1 REV	BGI	5'-GGCAAGCTACTTTTTTAAAGCA-3'
P2 FW	BGI	5'-GAGTGTCTGGCGTTCATGTTCC-3'
P2 REV	BGI	5'-AGAACATTCAATGCCCAGACAA-3'
Other		
Ampicillin	Sigma	Cat: BP021
Vancomycine	Sigma	Cat: V2002
Neomycin sulfate	Sigma	Cat: N6386
Metronidazole	Sigma	Cat: M3761
DMEM	Gibco	Cat:11965118
FBS	Gibco	Cat:10099141
HBSS	Gibco	Cat:14170161
Percoll	Solarbio	Cat: P8370
pcDNA™3.1/V5-His TOPO® TA Expression Kit	Invitrogen	Cat: K4800-40
Pierce™ Magnetic RNA-Protein Pull-Down Kit	Thermo Fisher Scientific	Cat:20164
DIG Northern Starter Kit	Roche	Cat:12039672910

Pierce™ Protein G Agarose	Thermo Fisher Scientific	Cat: 20397
LDH Assay Kit	Abcam	Cat: ab102526
Human Total bile acide ELISA Kit	Mybiosource	Cat: MBS723419
Human Deoxycholic Acid ELISA Kit	Mybiosource	Cat: MBS7254103
Human Lithocholic acid ELISA Kit	Mybiosource	Cat: MBS7273412

Table S2. **The predicting protein that bind with the *hu/mlncRNA57RIK* conservative sequence.**

Protein	Bioinformatic software programs	Predicted results
Caspase11	catRAPID，RPISeq，RNAInter	+++
Caspase4	catRAPID，RPISeq，RNAInter	+++
Hu-GBP1	catRAPID，RPISeq，	++
M-GBP1	catRAPID，RNAInter	++
+, indicates a potential binding of protein with lncRNA.		

Table S3. **The predicting protein binding sites in the *hu/mlncRNA57RIK* conservative sequence.**

Bioinformatic software	Number of caspase4 binding sites	Position	Matrix sequence
catRAPID	Three binding sites	2732bp-2746bp<br>2826bp-2837bp<br>2851bp-2870bp	CTGTTGTTGAAAAAT<br>AGGTTGTCTTTC<br>AACTGAAACTTGAGTTAGGG
RPISeq	Three binding sites	2717bp-2729bp<br>2812bp-2827bp<br>2846bp-2858bp	GCCATTTATATTT<br>ATGTAACTCCATTTAG<br>ACACTAACTGAAA
RNAInter	Two binding sites	2780bp-2793bp<br>2857bp-2871bp	TCAACAAAGTTGTT<br>AACTTGAGTTAGGGA
Bioinformatic software	Number of caspase11 binding sites	Position	Matrix sequence
catRAPID	Three binding sites	515bp-530bp<br>600bp-611bp<br>630bp-649bp	TTGCCATTTATGTGTT<br>AATAGAGTGGGA<br>GGTTTAGGTTGTCTTTTATT

RPISeq	Two binding sites	523bp-536bp<br>656bp-666bp	TATGTGTTTGTGTT<br>TACTGACATGA
RNAInter	Two binding sites	554bp-574bp<br>617bp-629bp	TTGATACCATAGGCATAGTTA<br>AGAAAGGTATTTA
Bioinformatic software	Number of Hu-GBP1 binding sites	Position	Matrix sequence
catRAPID	Three binding sites	2966bp-2980bp<br>3033bp-3045bp<br>3077bp-3097bp	AGGCAATGGTGTGAA<br>ATGTAATTGTATA<br>AACCCAGTCTTTGCTTTATGT
RPISeq	Two binding sites	3082bp-3102bp<br>3144bp-3156bp	AGTCTTTGCTTTATGTACAAA<br>AGTATGCTTTCAA
Bioinformatic software	Number of M-GBP1 binding sites	Position	Matrix sequence
catRAPID	Three binding sites	1259bp-1274bp<br>1380bp-1395bp<br>1438bp-1452bp	TACGATAGTCCACAGC<br>AAGCCAATTATTTGCT<br>TTGGAATAGAGAATG
RNAInter	Two binding sites	1325bp-1340bp<br>1406bp-1421bp	ATGATGAAAATGATTG<br>ATGCCTTGGTTCCTGA
